# A Bioengineered Mitochondrial Respiration‐Mimetic Copper‐Based Metal‐Organic Framework Reprogramming Oxidative Phosphorylation to Enhance Tumor Cuproptosis

**DOI:** 10.1002/advs.78669

**Published:** 2026-11-09

**Authors:** Liyang Zhou, Hongpei Deng, Tingli Zheng, Huan Min, Qiwei Tian, Huimin Lin, Jing Ruan, Xianqun Fan

**Affiliations:** ^1^ State Key Laboratory of Eye Health, Shanghai Key Laboratory of Orbital Diseases and Ocular Oncology, Department of Ophthalmology Ninth People's Hospital, Shanghai Jiao Tong University School of Medicine Shanghai People's Republic of China; ^2^ Henan Institute of Advanced Technology Zhengzhou University Zhengzhou People's Republic of China; ^3^ Shanghai Key Laboratory of Molecular Imaging Shanghai University of Medicine and Health Sciences Shanghai People's Republic of China

**Keywords:** cuproptosis, hybrid‐membrane, melanoma, mitochondrial metabolism, tumor targeting

## Abstract

Mitochondrial metabolism‐dependent cuproptosis has emerged as a promising anticancer modality, yet its efficacy is constrained by the hypoxic and glycolysis‐dominant tumor microenvironment that suppresses oxidative phosphorylation (OXPHOS). Strategies that simultaneously overcome hypoxia and mitochondrial metabolic resistance remain underexplored. Herein, we report a bioengineered mitochondrial respiration‐mimetic nanoplatform (TC@OMG) that renews tumor mitochondrial metabolism to enhance cuproptosis. TC@OMG integrates a copper‐based metal‐organic framework co‐loaded with glucose oxidase and a lactate dehydrogenase inhibitor, and is cloaked with a hybrid membrane derived from tumor cells and plant thylakoids, enabling tumor targeting and photosynthetic oxygen generation. Mechanistically, photo‐triggered TC@OMG couples hypoxia alleviation with glycolysis inhibition to enforce a metabolic shift toward mitochondrial OXPHOS, thereby sensitizing tumor cells to cuproptosis. Concurrently, its microenvironment‐responsive copper release and glutathione depletion disrupt mitochondrial Fe‐S cluster homeostasis and induce aggregation of lipoylated proteins, ultimately promoting cuproptotic cell death. In vivo, photo‐triggered TC@OMG achieves efficient tumor targeting, hypoxia relieving, and tumor inhibiting effects across multiple melanoma models, including subcutaneous, metastatic, and intraocular tumors. Collectively, this work bioengineered a respiration‐mimetic nanotherapeutic strategy that integrates mitochondrial metabolic reprogramming with copper‐dependent cell death, providing a generalizable strategy for overcoming mitochondrial metabolic resistance in cancer therapy.

## Graphical abstract

A bioengineered mitochondrial respiration‐mimetic nanoplatform (TC@OMG) renews tumor mitochondrial metabolism to enhance cuproptosis. TC@OMG integrates a copper‐based metal‐organic framework co‐loaded with glucose oxidase and a lactate dehydrogenase inhibitor, and is cloaked with a hybrid membrane derived from tumor cells and plant thylakoids, enabling tumor targeting and photosynthetic oxygen generation.

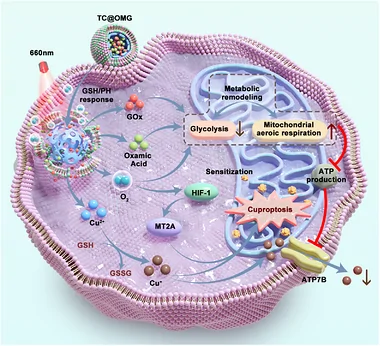

## 1 Introduction

Cuproptosis represents a recently discovered programmed cell death modality that mediates cytotoxicity through aggregated mitochondrial lipoylated proteins and destabilized iron‐sulfur cluster proteins [1]. Behind its biological mechanisms, the copper (Cu) ions concentration, intracellular oxygen content, and mitochondrial respiration activity all influence the tumor cuproptosis efficacy. Many studies have reported different strategies to trigger tumor cuproptosis, including of transporting external copper ions, inhibiting Cu‐efflux export and glutathione (GSH) biosynthesis, and upregulating the FDX1 expression [2, 3, 4, 5]. Our previous study firstly put forward regulating mitochondrial respiration strategy to sensitive cuproptosis [6]. However, under the hypoxic tumor microenvironment (TME), glycolysis could compensate for strong tumor energy consumption and make cuproptosis therapy escape [7, 8]. Thus, simultaneously inhibiting tumor cell glycolysis and alleviating the hypoxic microenvironment is imperative for enhancing cuproptosis.

To address the Warburg effect [9], glucose oxidase (GOx), a potent glucose catalytic agent, was reported to not only exhaust the glycogen supply [10], but also promote mitochondrial oxidative phosphorylation through the p53 pathway and NF‐κB pathway [11, 12, 13, 14]. However, GOx‐based tumor starvation therapeutic efficacy was limited by hypoxia TME, short half‐life, nonspecific systemic toxicity, and insufficient catalytic efficiency [15, 16]. Oxamic acid functions as a key pyruvate metabolic modulator that specifically inhibits lactate dehydrogenase (LDH‐A) activity and suppresses lactic acid production [17]. Tumor cells exhibit increased LDH activity under the Warburg effect, indicating that supplementing oxamic acid would proactively inhibit lactate production and promote the pyruvate dedicated to the tricarboxylic acid (TCA) cycle [18, 19]. However, the practical application of free oxamic acid is constrained by its high hydrophilicity that limits cellular uptake efficiency and leads suboptimal therapeutic outcomes when administered as a single agent. Meanwhile, cancer cells exhibit remarkable metabolic plasticity, enabling them to rapidly activate compensatory pathways that circumvent LDH‐A inhibition [20, 21]. To overcome these limitations, the engineered nanoplatform could overcome the small drugs biodistribution problem [22], and even benefit from co‐delivering two kinds of drugs, GOx and oxamic acid.

To effectively ameliorate hypoxic microenvironment, inspired by the thylakoid (TK), a fundamental chloroplast substructure with a stacked membrane architecture, acts as natural photo‐driven oxygen production microscopic factory, offering a built‐in photosynthetic engine for continuous oxygen supply [23]. The peculiar photocatalytic water‐splitting property enable TK to achieve in‐situ oxygen production, which is expected to help alleviate tumor hypoxia [24]. Previous studies have reported the TK‐engineered membranes have good tissue repairment and anti‐inflammatory function in hypoxia and ischemia physiological environment, and can enhance mitochondrial metabolism [23]. However, this biomimetic strategy remains unexplored for tumor therapy [25, 26]. In addition, plenty of oxygen supply could also enable intracellular Cu ions participated into the mitochondria metabolism [27]. Consequently, using the TK membrane to modify nanoparticle could address the cuproptosis therapeutic's hypoxia TME [28]. Additionally, considering the nanodrug's the systematic non‐specific toxicity, tumor cell membrane coating technology could acquire homologous targeting capability by preserving antigenic and adhesive profiles of native tumor cell, thus dual membrane hybrid‐fused strategy would benefit for the tumor hypoxia alleviation and targeting therapy [29, 30].

Apart from ameliorating the special tumor environment, sufficient intracellular copper ions are another necessary condition to trigger cuproptosis. Monovalent copper ions (Cu^+^) are the core executors of cuproptosis, which can induce abnormal oligomerization of thiocylated proteins (such as dihydrolipoamide S‐acetyltransferase, DLAT) in the TCA cycle, disrupt the stability of iron‐sulfur cluster proteins, and inhibit the ubiquitin‐proteasome system [31, 32]. Owing to the copper‐based metal‐organic framework (MOF) specific advantages, such as drug‐carrying capacity, cell internalization ability, tuneability, and recyclability, MOFs exhibit good application potentials in inducing tumor cuproptosis [33, 34, 35]. However, the efficacy of current strategies is often constrained by the complex tumor microenvironment (TME) [36], necessitating the optimization of MOF design. Therefore, the ideal bioengineered MOF system design should achieve efficient release of Cu^+^ or in situ reduction of Cu^2^^+^ to Cu^+^, while synergistically overcoming the limitations of the TME (such as high GSH, hypoxia, acidification, oxidative stress, etc.), positioning the bioengineered Cu‐MOF as a potent cuproptosis therapeutic agent.

Herein, we reported a hybrid membrane bio‐engineered metal‐organic framework (MOF)‐based nanodrug (TC@OMG), in which oxamic acid and GOx are conjugated into the MOF by chemical conjugation and physical adsorption to form the OMG core, and then the OMG was further encapsulated by a hybrid membrane derived from tumor cells and thylakoids to yield the nanodrug TC@OMG. Through the photosynthetic effect, the prepared nanodrug could effectively ameliorate hypoxic TME and inhibit glycolysis, which remodels the tumor cell metabolism into mitochondrial OXPHOS, finally enhancing tumor cuproptosis efficiency (Figure 1a). This study established a bioengineered respiration‐mimetic nanodrug to enhance tumor cuproptosis through reprogram mitochondrial respiration.

**FIGURE 1:**
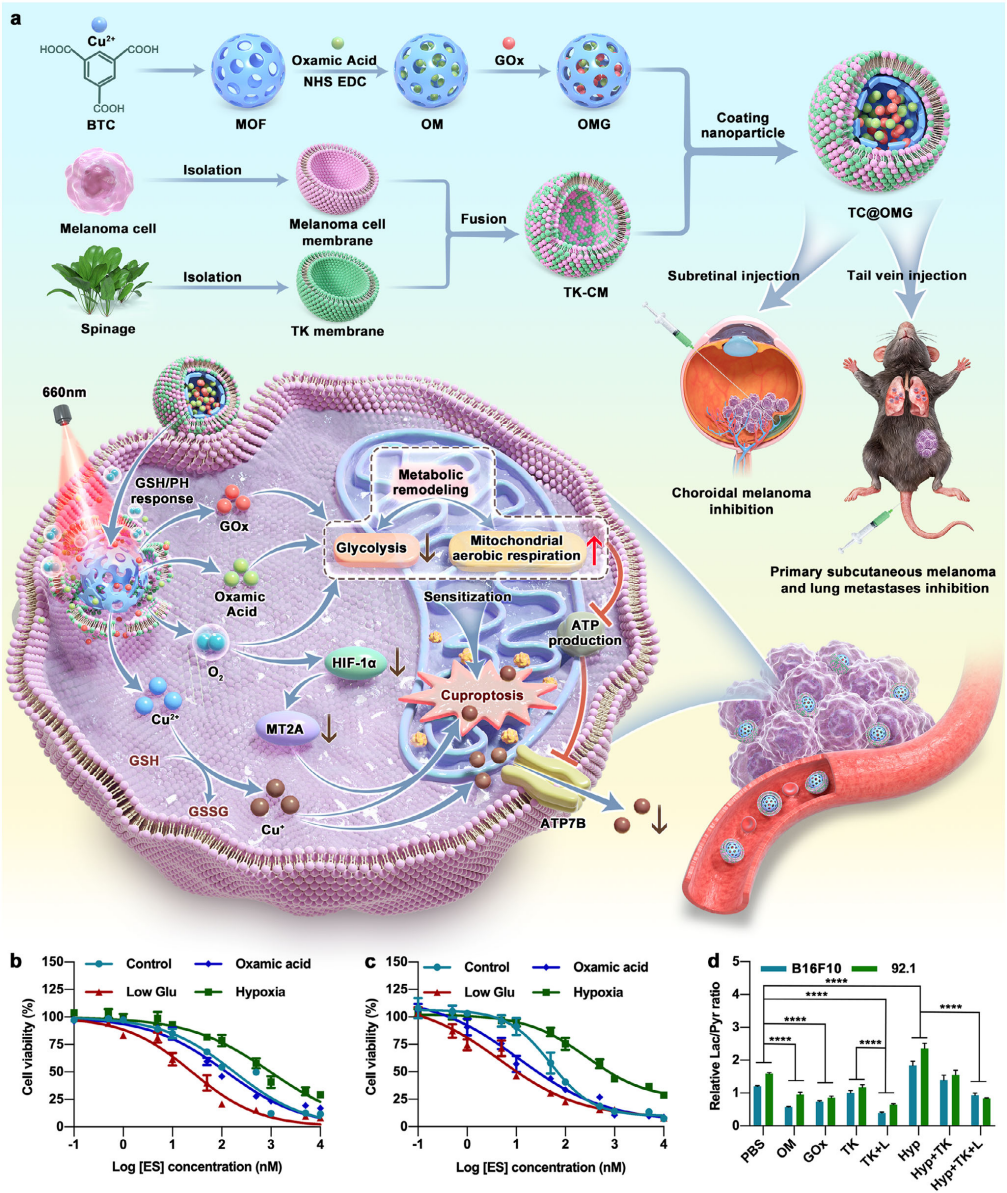
Schematic diagram of TC@OMG‐mediated mitochondrial metabolic reprogramming and cuproptosis sensitization in tumor cells. (a) Preparation and action mechanism of TC@OMG. (b) Typical dose‐response curves of ES before and after treatment in B16F10 and (c) 92.1 cells, *n* = 5. (d) The lactate/pyruvate ratio of B16F10 and 92.1 cells before and after treatment. The measurement was obtained based on the ratio of [lactic acid] to [pyruvate]. Glu, glucose; GOx, glucose oxidase; TK, thylakoid; L, laser; Hyp, hypoxia. Data are presented as mean ± SD, *n* = 3, significance is determined by two‐way ANOVA with Tukey's correction. Significance levels are denoted as follows: *p* < 0.05(*), *p* < 0.01(**), *p* < 0.001(***), and *p* < 0.0001(****); ns, no significance.

## 2 Results and Discussion

### 2.1 Cuproptosis Therapeutics Resistance to the Tumor Metabolic Microenvironment

To directly validate the hypothesis that enhanced mitochondrial metabolism sensitizes cells to cuproptosis, the cuproptosis inducer ES (Elesclomol) was systematically evaluated under various metabolic perturbations, including low glucose, hypoxia, and LDH‐A inhibition. Under the low glucose and LDH‐A conditions, the B16F10 cells half‐maximal inhibitory concentration (IC50) decreased to 25 and 123 nM, respectively, from the baseline of 179 nM. In contrast, under hypoxic conditions, the IC50 increased to 971 nM (Figure 1b). Consistent results were obtained in 92.1 cells, confirming that the coordination of oxygen enrichment, glucose restriction, and LDH‐A inhibition constituted a promoting factor for enhancing the efficiency of cuproptosis (Figure 1c).

To further elucidate the differential response of B16F10 and 92.1 melanoma cells to ES treatment, the lactate/pyruvate ratio in the cells was assessed following various metabolic perturbations (Figure 1d and Figures S1 and S2). Compared with the normal control (PBS) group, glucose oxidase and oxamic acid treated cells exhibited significantly reduced lactate/pyruvate ratio, implying an impaired Warburg effect. Conversely, single hypoxic group cells showed a significant increase in the lactate/pyruvate ratio. Interestingly, this ratio was reduced after hypoxia ameliorated by the TK‐mediated photosynthetic oxygenation, demonstrating effective mitigation of the hypoxic metabolic shift. Notably, the decline in the lactate/pyruvate ratio was paralleled by an increased ES sensitivity of cells, underscoring a strong correlation between the metabolic profile and the cuproptosis susceptibility of tumor cells.

### 2.2 Preparation and Characterization of TC@OMG

The bioengineered mitochondrial respiration‐mimetic nanodrug TC@OMG was fabricated. In detail, the Cu‐based MOF (Cu‐MOF) was firstly constructed through coordinating Cu^2+^ and benzene‐1,3,5‐tricarboxylic acid (BTC). Subsequently, GOx and oxamic acid were co‐encapsulated within the Cu‐MOF framework (OMG) through a combination of free diffusion and amide bond reaction. Finally, the Cu‐MOF was subsequently functionalized through encapsulation with a hybrid biomembrane of TK and tumor cell membranes. All the intermediate products showed different morphological changes, as shown in Figure S3.

The MOF shows specific Fourier transform infrared spectroscopy (FT‐IR) and X‐ray diffraction (XRD) spectrums as previously reported (Figures S4 and S5) [37]. As shown in Figure 2a and Figure S6, transmission electron microscopy (TEM) and scanning electron microscope (SEM) characterization revealed monodisperse spherical morphology of the synthesized MOF nanoparticles. Dynamic light scattering (DLS) measurements indicated a hydrated particle size of approximately 100.39 nm for MOF, with a corresponding zeta potential (ζ) of +12.07 mV (Figure 2c,d). The as‐synthesized TC@OMG zeta potential showed −3.58 mV compare with the −17.59 mV of TK‐CM (TC) hybrid membrane and +11.70 mV of OMG, which illustrated that MOF was effectively encapsulated and surface modified with TK‐CM hybrid membranes (Figure 2d). TEM and SEM images clearly revealed that TC@OMG has good dispersity with an average diameter of 260 nm (Figure 2b and Figure S7), which is consistent with the DLS results (Figure 2c). The intermediate product of OMG was confirmed by detection of nitrogen in X‐ray photoelectron spectroscopy (XPS) spectra and ^1^H MR spectrum (Figure 2e and Figures S8–S10). Elemental analysis (EA) of nitrogen content increased from 0.73 wt.% (pristine MOF) to 9.55 wt.% (OM), confirming successful oxamic acid conjugation. Further calculation revealed that the oxamic acid loading was approximately 5.61 mmoL/g MOF (Figure S11). In the XPS spectrum, two characteristic peaks assigned to Cu^2+^2p_3/2_ (936.6 eV) and Cu^2+^2p_1/2_ (955.8 eV) were observed, confirming the dominant presence of Cu^2^^+^ in the MOF (Figure S12). N_2_ adsorption‐desorption isotherm analysis confirmed the retention of GOx adsorption capacity in MOF following conjugation with oxamic acid (Figure S13). The characteristic chlorophyll absorption peak at 640–660 nm, originating from thylakoid membranes, was distinctly observed in both TK‐CM membrane and TC@OMG nanoparticles by UV‐vis spectroscopy, verifying successful membrane coating (Figure 2f). Elemental mapping of TC@OMG confirmed the homogeneous spatial distribution of carbon, oxygen, nitrogen, and Cu throughout the nanostructure (Figure 2g). The encapsulation efficiency and loading capacity of GOx in MOF and OM were quantitatively analyzed using the G250 probe (Figure S14). At a GOx to MOF feeding ratio of 50%, the encapsulation efficiency was 52.58%, and the encapsulation capacity was 21.02%. At a feeding ratio of GOx to OM of 50%, the encapsulation efficiency was 58.69%, and the encapsulation capacity was 22.67%, indicating no significant alteration in the modified OM compared to MOF (Figures S15 and S16). The hybrid membranes fusion effect was observed from the fluorescent microscopy. It shows a distinct yellow signal instead of the physical mixture of fluorescence signals (Figure S17). The thermogravimetric analysis (TGA) results illustrated that the TC@OMG mass loss rate is slower than the OMG group, indicating the effective encapsulation of TK membrane (Figure S18). Moreover, sodium dodecyl sulfate‐polyacrylamide gel electrophoresis (SDS‐PAGE) confirmed the presence of TK and tumor cells membrane‐specific proteins in TC@OMG (Figure 2h).

**FIGURE 2:**
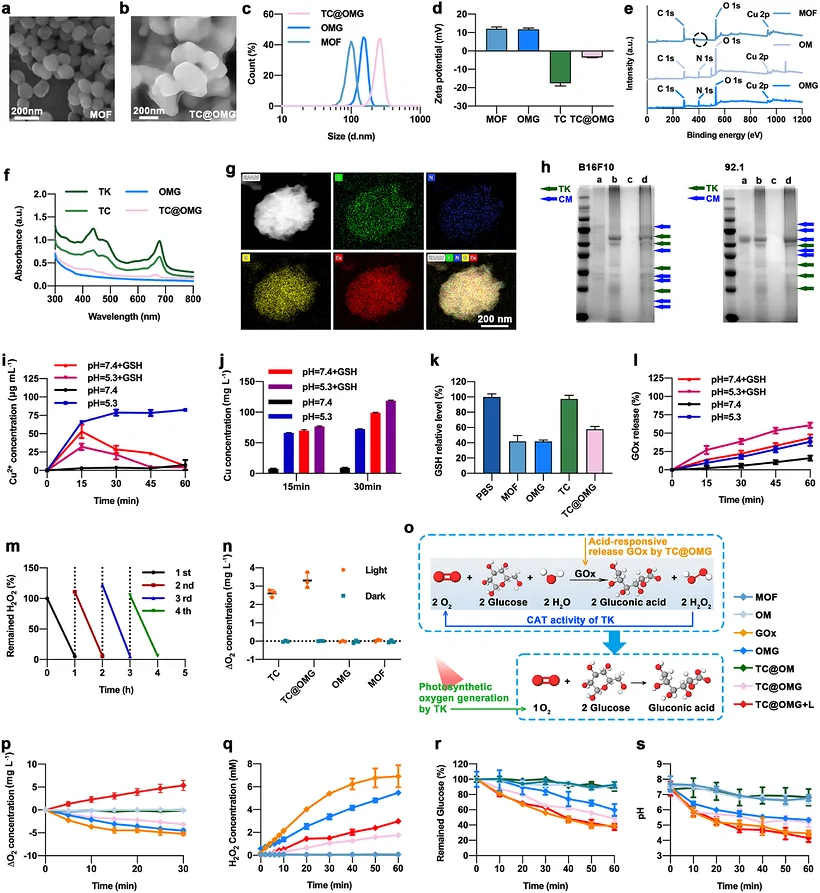
The characterization of TC@OMG. (a) SEM images of the MOF and (b) TC@OMG, Scale bars = 200 nm. (c) Size distribution analysis of TC@OMG, OMG, and MOF. (d) Zeta potential analysis of the MOF, TC, OMG, and TC@OMG. (e) XPS spectra analysis (C, N, O, and Cu elements) of MOF, OM, and OMG. (f) UV‐vis absorbance spectra of TK, TC, OMG, and TC@OMG. (g) Element mappings including C, N, O, and Cu of TC@OMG. Scale bar = 200 nm. (h) SDS‐PAGE showing the membrane proteins presence after TK membrane fusion with B16F10 and 92.1 cell membrane. a: cell membrane; b: TK membrane; c: MOF; d: TC@OMG. (i) Cu^2+^ release by TC@OMG in 60 min. (j) Acid‐ and GSH‐responsive total Cu release in 30 min. (k) Residual GSH levels after Cu^2+^‐mediated GSH depletion. (l) Acid‐ and GSH‐responsive GOx release. (m) Repeated H_2_O_2_ cyclic catalysis 4 times. (n) Oxygen production of MOF, OMG, TK‐CM, and TC@OMG after 660 nm laser irradiation for 10 min. (o) Schematic diagram of the GOx cascade reaction. (p) Oxygen concentration during GOx catalysis in 30 min. (q) H_2_O_2_ concentration, (r) glucose, and (s) pH change after the GOx cascade reaction in 60 min. Data are presented as mean ± SD, *n* = 3.

### 2.3 Acid and GSH Dual‐Responsive Drug Release

The copper ions release and valence transition were evaluated using sodium diethyldithiocarbamate (DDTC) as a colorimetric chelator for Cu^2+^ detection (Figure S19). Cu ion release from the MOFs demonstrated clear acid‐ and GSH‐dependent characteristics (Figure 2i and Figure S20). TC@OMG exhibited minimal Cu^2+^ release (≤6.08 µg mL^−1^) after 60 min at a pH of 7.4, while under acidic conditions (pH 5.3), Cu^2+^ showed time‐dependent release enhancement. When both acidity (pH 5.3) and GSH (10 mM) were present, the Cu^2+^ concentration peaked at 15 min (32.19 µg mL^−1^) followed by a progressive decrease, confirming the GSH‐mediated reduction of Cu^2+^ to Cu^+^. The further Cu^+^ release results proved that its concentration reached maximum only when GSH existed, regardless of pH (Figure S21). Inductively coupled plasma (ICP) analysis confirmed that the total Cu ion release reached its maximum level of 118.87 mg L^−1^ at 30 min under coexistence of acidic and GSH (Figure 2j). Both the acid‐responsive release of Cu^2+^ and the GSH‐mediated generation of Cu^+^ from TC@OMG exhibited clear concentration‐dependent profiles (Figures S22 and S23). Noticeably, both Cu‐MOF and OMG demonstrated significant GSH depletion effects (Figure 2k), while the GSH level reversed a lot after medicated with thylakoid membrane in a concentration‐dependent manner (Figure S24).

Subsequently, the cargo release behavior of MOF was examined. The in vitro GSH‐ and acid‐responsive release profiles of GOx from MG and TC@OMG were evaluated (Figure 2l and Figure S25). The results showed that both MG and TC@OMG exhibited acid‐ and GSH‐dependent GOx release profiles. Specifically, under normal conditions (pH 7.4, 0 mM GSH), TC@OMG demonstrated minimal GOx leakage (≤15.98%). When exposed to acidic buffer (pH 5.3) for 60 min, the GOx cumulative release was significantly enhanced and reached 38.28%, while upon the introduction of 10 mM GSH, GOx was elevated to 60.95%. Especially, TC membrane encapsulation could significantly suppress nonspecific GOx release and maintained its responsive release capability in acidic/GSH‐rich microenvironments. These findings fully suggested that TC@OMG has dual‐responsive drug release characteristics, and the acid and GSH microenvironments could initiate the subsequent GOx‐catalyzed cascade.

### 2.4 Catalase (CAT) and Photosynthesis

The encapsulation of TK‐CM conferred upon TC@OMG the ability to catalyze H_2_O_2_, because the TK membrane has strong photosynthetic oxygen generation capability and CAT‐like activity. Meanwhile, the CAT‐like activity of hybrid membranes is varied from the different TK:CM ratios, the H_2_O_2_ consumption capacity exhibited a positive correlation with the thylakoid membrane proportion, and the 1:1 ratio has a moderate effect (Figure S26). Even the TC@OMG shows similar CAT‐like activity as TC hybrid membranes (Figure S27). Furthermore, the stability of the catalase‐like activity in TC@OMG was also evaluated through four consecutive catalytic cycles, each comprising 1‐hour incubation with 1 mM H_2_O_2_. Comparable H_2_O_2_ depletion rates across all cycles confirmed the robust and sustainable catalytic performance of the nanoplatform (Figure 2m). To further quantify the CAT‐like activity of TC@OMG, the oxygen production capacity of all the groups was evaluated. The results illustrated that TC@OMG has similar effect as hybrid TC membranes and intermediate between TK and MOF groups (Figure S28). In addition, the TK/CM ratio with 1:1 exhibited a moderate oxygen production effect (Figure S29). The above results proved that the 1:1 ratio of TK/CM encapsulated TC@OMG nanoparticles has good CAT and photosynthesis roles and were used in the following experiments. Notably, TC@OMG exhibited enhanced photosynthetic efficiency by producing 3.22 mg L^−1^ O_2_ under 660 nm laser light irradiation (Figure 2n) due to its TK membrane (Figure S30), and the O_2_ generation ability acts as a dosage (Figure S31) and laser flux dependent manners (Figure S32). These findings proved TC@OMG has the integrated capabilities of efficient H_2_O_2_ catalysis coupled with sustained photosynthetic oxygen generation, which can directly address the dual pathological challenges of hypoxia and oxidative stress in the tumor microenvironment.

### 2.5 GOx Cascaded Catalysis and Photosynthetic Effects

As the primary substrate for cellular energy metabolism, glucose starvation can effectively suppress tumor energy supply while inducing metabolic reprogramming toward oxidative phosphorylation. For TC@OMG nanodrug, the tumor acidic microenvironment promoted subsequent GOx release, while H_2_O_2_ was catalytically decomposed to O_2_ via the CAT‐like activity of TK membranes, thereby establishing a self‐sustaining catalytic cycle. Notably, each catalytic turnover consumed two glucose molecules per oxygen molecule, collectively depleting intratumoral glucose reserves (Figure 2o). Crucially, oxygen replenishment was dually maintained through both H_2_O_2_ decomposition and continuous photosynthetic production under light irradiation, ensuring stable operation of the entire glucose metabolism. To determine whether GOx‐catalyzed O_2_ consumption could compromise the overall oxygenation efficacy, dissolved O_2_ levels were monitored among different nanodrug groups. MOF showed no O_2_ consumption or production, whereas OMG (GOx‐loaded Cu‐MOF) exhibited significant oxygen depletion. In contrast, TC@OMG partially compensated for this oxygen demand and achieved a significant increase in O_2_ concentration when synergetic with light irradiation (TC@OMG+L) (Figure 2p). These results demonstrated that the photosynthetic functionality of TC@OMG counterbalanced GOx‐induced oxygen depletion and effectively alleviated hypoxia TME. To assess the TK membranes CAT effect on the microenvironment H_2_O_2_ levels, post‐reaction H_2_O_2_ concentrations were quantified among different groups. While GOx catalysis generated substantial H_2_O_2_, TC@OMG with the TK membrane significantly reduced the H_2_O_2_ accumulation (Figure 2q). It confirmed the dual function of the TK membrane in decomposing GOx‐derived H_2_O_2_ while replenishing oxygen, thereby establishing a self‐accelerating catalytic cycle. This glucose catalyze reaction efficiency was further evaluated through glucose consumption and pH variation, the results showed that neither MOF nor OM exhibit glucose degradation capability, whereas TC@OMG demonstrated rapid glucose depletion under light irradiation (TC@OMG+L) (Figure 2r). Correspondingly, the solution pH in the TC@OMG+L group progressively decreased from 7.4 to 4.15 within 60 min, consistent with gluconic acid generation (Figure 2s). In contrast, the MOF and the OM group maintained neutral pH throughout the experiment, confirming the essential role of photosynthetic activating GOx in driving the acidic product accumulation. To determine whether gluconic acid production establishes a positive feedback loop with the acid‐responsive dissociation of TC@OMG, the release profiles of GOx (Figure S33), Cu^2+^ (Figure S34), and Cu^+^ (Figure S35) were compared under glucose‐supplemented and glucose‐free conditions. Both GOx and Cu^2+^ displayed glucose‐dependent release kinetics, whereas Cu^+^ concentrations remained stable. These findings confirm that glucose‐triggered MOF dissociation, mediated by gluconic acid generation, enables controlled drug release from TC@OMG. Integrating GOx‐mimetic and CAT‐like activities, TC@OMG+L enabled concurrent glucose decomposition and H_2_O_2_ scavenging through synergistic cascade catalysis and photosynthetic oxygen evolution. This dual‐pathway mechanism effectively alleviated tumor hypoxia while inducing further glucose starvation‐mediated metabolic suppression.

### 2.6 Evaluation of TC@OMG Efficacy and Biocompatibility

The cytotoxicity of various MOF‐based formulations was evaluated in B16F10 and 92.1 cell lines. At concentrations exceeding 40 µg mL^−1^, TC@OMG+L exhibited significantly enhanced cytotoxicity against both B16F10 (Figure 3a) and 92.1 (Figure 3b) cell lines (22.16% and 31.04% cell viabilities), representing a substantial decrease compared to the MOF group. Component functional analysis demonstrated that MG at 40 µg mL^−1^ significantly suppressed cell viability, reducing survival rates by 39.41% (B16F10) and 32.05% (92.1) compared to the MOF control. OM treatment (40 µg mL^−1^) reduced B16F10 and 92.1 cell viability by 17.95% and 13.52%, respectively, compared to the MOF control group. TC@M treatment (40 µg mL^−1^) exhibited moderate cytotoxicity with cell viability rates of 56.15% (B16F10) and 62.09% (92.1) after 24 h of incubation. Following 660 nm irradiation, cell viability further decreased to 45.97% and 52.85%, respectively, demonstrating light‐enhanced therapeutic efficacy. Collectively, the photosynthetic bio‐hybrid system integrating MOF, GOx, oxamic acid, and TC membrane demonstrated limited cytotoxicity alone, while amplified synergistic cytotoxicity cooperated (Figures S36–S38). Pronounced cytotoxicity against B16F10 cells was confirmed by calcein‐AM/PI dual staining for TC@M+L, MG, and TC@OMG+L, whereas minimal cytotoxicity was observed in the TC@M, OM, and MOF group (Figure S39). The remarkable anti‐proliferative effect of TC@OMG+L on B16F10 cells was further verified by crystal violet staining (Figure S40). To validate the homologous targeting capability of the TC membrane, quantitative fluorescence analysis of cellular uptake was conducted to compare TC@M with MOF across different cell lines (Figure 3c,d). Significantly enhanced cellular uptake of TC@M coated with a homologous cancer cell membrane was observed in B16F10 and 92.1 cells compared to MOF, whereas reduced internalization was demonstrated in heterologous PIG1 and RPE cells (Figure 3e). The endocytosis of different hybrid membrane ratios by tumor cells was assessed using FITC. As illustrated in Figure 3f, TC@OMG featuring a hybrid membrane with a higher proportion of tumor cell‐derived components was found to significantly enhance cellular uptake, leading to improved therapeutic outcomes. Notably, after the TC membrane encapsulation, the TC@M exhibited IC50 values of 22.84 and 7.27 µg mL^−1^ in RPE cell and PIG1 cell lines respectively (Figures S41 and S42). Even though under treating with 0.8 µg mL^−1^ of TC@OMG, both RPE and PIG1 cell lines maintained a survival rate of over 80% (Figure S43). Meanwhile, the cytotoxicity of the TC membrane itself is relatively limited (Figure S44). This differential uptake pattern confirmed the tumor‐targeting specificity conferred by TC membrane modification, which improved its bioavailability. Hemolysis assays demonstrated the good blood compatibility of TC@OMG (Figures S45 and S46). The above experimental evidence fully established that TC@OMG+L selectively inhibited tumor cell survival and proliferation while exhibiting substantially lower cytotoxicity to normal cells.

**FIGURE 3:**
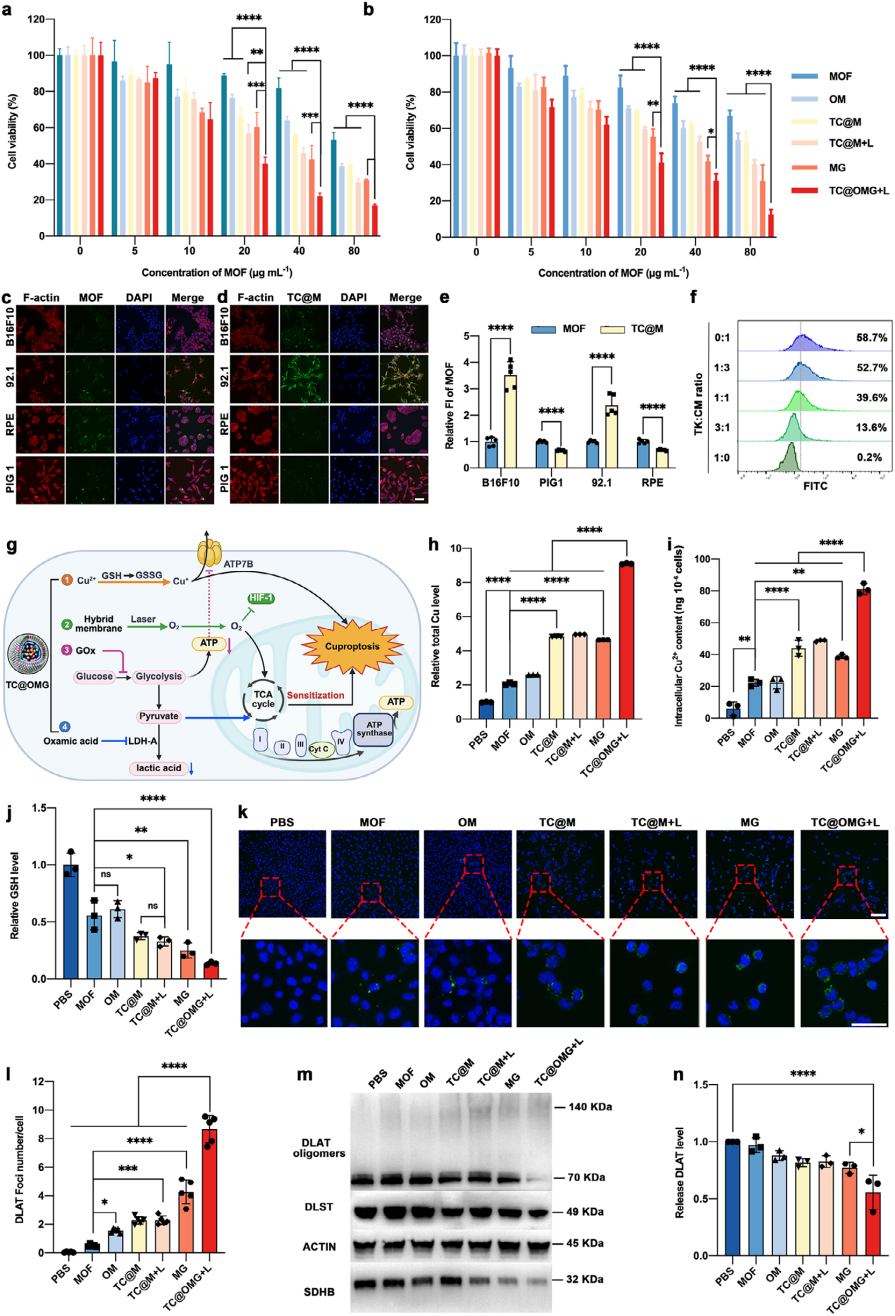
In vitro Tumor cell killing and cuproptosis induction mechanisms of TC@OMG. (a) Relative viabilities of B16F10 and (b) 92.1 cells after incubation with TC@OMG at various concentrations for 24 h. Significances are determined by two‐way ANOVA with Tukey's correction. (c) Representative fluorescence confocal images to evaluate cellular uptake of MOF and (d) TC@M by B16F10, PIG1, 92.1, and RPE cells and e) the quantification. Significances are determined by paired *t*‐test. Scale bar = 100 µm. (f) Representative flow cytometry plots showing internalization of TC@OMG with different TK:CM ratios in B16F10 cells. (g) Schematic diagram of TC@OMG‐reprogrammed B16F10 metabolism and sensitized cuproptosis. Image was created with BioRender.com (Agreement number: 9FDJ7149JN). (h) Relative intracellular total Cu, (i) Cu^2+^, and (j) GSH contents of B16F10 cells after different treatments. (k) Representative immunofluorescence images of DLAT oligomers (green foci) and enlarged images of B16F10 cells after different treatments. Scale bar = 100 µm, respectively. (l) Quantitative analysis of the number of DLAT foci per B16F10 cell after different treatments. (m) Western blot images of DLAT oligomers DLST and Fe‐S cluster proteins (SDHB) relative to ACTIN in B16F10 cells after different treatments. (n) Relative DLAT activity in B16F10 cells after different treatments. Data are presented as mean ± SD, *n* = 3. Significance is determined by one‐way ANOVA with Tukey's correction. Significance levels are denoted as follows: *p* < 0.05(*), *p* < 0.01(**), *p* < 0.001(***), and *p* < 0.0001(****); ns, no significance.

The biocompatibility of TC@OMG was further systematically evaluated in vivo. Biochemical analysis of blood samples collected 12 days after injection revealed comparable levels of hepatic (ALP, AST, ALT) and renal (creatinine, urea) function markers across PBS, MOF, and TC@OMG groups, with no body weight change (Figures S47a,b and S48). Histopathological examination further confirmed the absence of significant morphological alterations or inflammatory lesions in major organs following MOF or TC@OMG administration compared to PBS controls (Figure S47c). These comprehensive analyses establish the excellent biosafety profile of TC@OMG.

### 2.7 TC@OMG Sensitizes Tumor Cuproptosis

This study developed a multimodal strategy to potentiate cuproptosis by remodeling the TME through four coordinated mechanisms: (1) Provides exogenous copper ions donors that act both as a GSH oxidant and as an initiator of cuproptosis; (2) Through photosynthesis, the TK‐functionalized hybrid membrane produces oxygen to relieve hypoxia, driving mitochondrial respiration shift in tumor cells and potentiating copper‐mediated oxidative stress; (3) GOx‐mediated catalytic depletion of glucose to induce energy crisis; (4) Oxamic acid‐mediated inhibition of LDH‐A to redirect glycolytic flux toward mitochondrial metabolism. These synergistic modalities integration established a promising metabolic landscape for cuproptosis execution through the disruption of intracellular copper homeostasis, achieving a potent and selective tumor killing effect (Figure 3g).

Cuproptosis progression was regulated by the dynamic antagonism between intracellular GSH and Cu ions, where Cu functioned as the executioner by disrupting mitochondrial respiration, while GSH served as the inhibitor by chelating Cu^2+^ and maintaining redox homeostasis. Treatment with TC@OMG+L significantly depleted GSH reserves while simultaneously elevating intracellular Cu^2+^ and total Cu levels (Figure 3h–j). The disruption of GSH and Cu^2+^ balance coordinated trigger cuproptosis in tumor cells.

Subsequently, the mechanism underlying cuproptosis was further analyzed.DLAT serves as a key cuproptosis target, which lipoylated sites directly bind to Cu ions to induce abnormal oligomerization. The DLAT oligomerization triggers mitochondrial metabolic dysfunction and proteotoxic stress, ultimately leading to tumor cell death. The immunofluorescent staining results demonstrated that MOF, OM, TC@M, TC@M+L, and MG treatments all elevated DLAT oligomer levels compared to the PBS group. The most substantial increase was observed in TC@OMG+L treated B16F10 cells, indicating significantly enhanced cuproptosis induction (Figure 3k,l). Consistent with these findings, western blot analysis confirmed enhanced DLAT oligomerization in the TC@OMG+L group, accompanied by the loss of DLST and downregulated Fe‐S cluster protein SDHB (Figure 3m,n). The downregulated expression of mitochondrial respiratory chain complex I of NDUFB8 and upregulated cuproptosis key effector protein of FDX1 synergetic illustrating cuproptosis enhanced and mitochondrial function decreased (Figure S49). These results collectively demonstrated that TC@OMG+L significantly amplified cuproptosis efficacy.

The efficacy of hybrid membrane‐mediated photosynthetic oxygenation in modulating hypoxic TME was also assessed (Figure 4a–d). Significantly enhanced RDPP and HIF‐1α fluorescent signals were observed in the MG group, confirming GOx‐mediated oxygen consumption. In contrast, TC@OMG+L treatment substantially decreased both RDPP and HIF‐1α signals, demonstrating that it effectively replenish oxygen consumed. Further analysis revealed that the oxygen produced through photosynthesis played a major role in this process (Figure S50). Cell intracellular oxidative stress was further evaluated following various treatments. A significant increase in total ROS levels was exclusively observed in the MG group, whereas other groups showed no notable changes, including PBS controls (Figure 4e,f). Parallel measurements of H_2_O_2_ also revealed a specific elevation only in the MG and GOx group, confirming that ROS accumulation mainly originated from GOx‐catalyzed H_2_O_2_ generation (Figure 4g and Figure S51), hinting that the GOx‐mediated cascade reaction was effective in tumor cells. Finally, the mitochondrial membrane potential (ΔΨm) was assessed post TC@OMG+L treatment. After 24‐hour incubation, TC@OMG+L significantly depolarized cells ΔΨm and intensified mitochondrial damage (Figure 4h,i). This finding aligns with the DLAT oligomerization results. This pathological depolarization was suspected to be cuproptosis‐mediated and a precursor of mitochondrial dysfunction.

**FIGURE 4:**
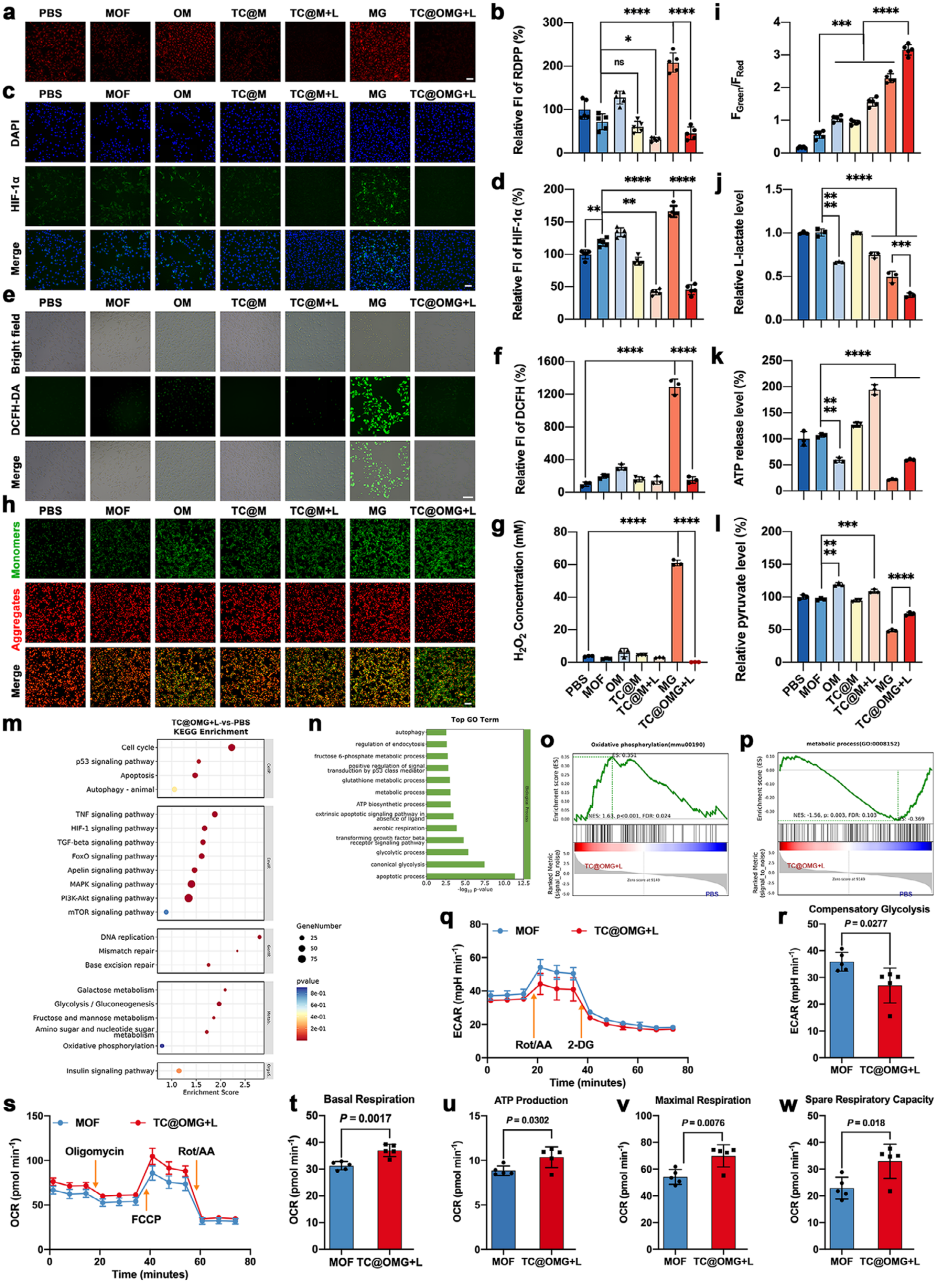
TC@OMG reprograms mitochondrial respiration. (a) Intracellular hypoxia levels and (b) quantification using the RDPP probe after different treatments. Scale bar = 100 µm. (c) HIF‐1α immunofluorescence staining and (d) quantification after different treatments. Scale bar = 100 µm. (e) Intracellular ROS levels detected by the probe DCFH‐DA after different treatments and (f) corresponding quantitative analysis. Scale bar = 100 µm. (g) Intracellular H_2_O_2_ levels detected after different treatments. (h) Mitochondrial membrane potential and (i) quantification using the JC‐1 probe after different treatments. Scale bar = 100 µm. (j) Intracellular L‐lactate levels, (k) ATP levels, and (l) pyruvate levels after different treatments. Data are presented as mean ± SD, *n* ≥ 3. Sigificance is determined by one way ANOVA with Tukey's correction. (m) KEGG enrichment bubble plot of the enriched pathways between B16F10 cells treated with TC@OMG+L and those treated with PBS (fold change ≥1.5, *p* < 0.05). (n) GO enrichment bubble plot of the enriched pathways between B16F10 cells treated with TC@OMG+L and those treated with PBS (fold change ≥ 1.5, *p* < 0.05). (o) GSEA showing that the mountain plot for oxidative phosphorylation and (p) metabolic process hallmark genes were enriched in B16F10 cells treated with TC@OMG+L. (q) Dynamic curve of extracellular acidification rate (ECAR) and (r) compensatory glycolysis of B16F10 cells treated with TC@OMG+L or MOF. (s) Oxygen consumption rate (OCR) in B16F10 cells treated with TC@OMG+L or MOF. (t) Basal respiration, (u) maximal respiration, (v) ATP production, and (w) spare respiratory capacity of B16F10 cells treated with TC@OMG+L or MOF. Data are presented as mean ± SD, *n* = 5. Significance is determined by a paired *t*‐test. Significance levels are denoted as follows: *p* < 0.05(*), *p* < 0.01(**), *p* < 0.001(***), and *p* < 0.0001(****); ns: no significance.

### 2.8 TC@OMG Reprogramming Hypoxia TME to Enhance Mitochondrial Respiration

Following the analysis of the apoptotic status, we further assessed the cellular metabolic state alteration by detecting key metabolites of glucose metabolism. The results showed that OM, MG, and TC@OMG+L treatments reduced glucose availability and inhibited LDHA, leading to decreased ATP and lactate along with pyruvate accumulation (Figure 4j–l, Figures S52 and S53). The combined action of GOx and oxamic acid effectively suppressed aerobic glycolysis in B16F10 cells by targeting both substrate supply and a key enzyme (Figure S54). This created a metabolic state favoring oxidative phosphorylation, while the induced cellular starvation further promoted this shift toward efficient glucose utilization.

Glucose metabolic pathway alterations under different treatments were evaluated through transcriptomic analysis, along with glycolysis and mitochondrial respiration assessments. Correlation and principal component (PCA) analyses revealed high intra‐group transcriptional consistency, with maximal inter‐group divergence between the TC@OMG+L and negative control groups (Figures S55 and S56). RNA‐sequencing identified 2262 upregulated and 1805 downregulated genes in the TC@OMG+L group (Figure S57). Kyoto Encyclopedia of Genes and Genomes (KEGG) and Gene Ontology (GO) analyses demonstrated significant enrichment in metabolic pathways and autophagy pathway, while Gene Set Enrichment Analysis (GSEA) confirmed global metabolic suppression concurrent with enhanced oxidative phosphorylation (Figures 4m–p and S58). Functional analysis of the TC@OMG+L group revealed distinct mechanisms for OMG and TC@M+L. OMG significantly enriched autophagy, apoptosis, and cell cycle arrest pathways through KEGG and GO analyses compared with the PBS group, indicating metabolic reprogramming to improve cellular fitness and survival during glucose deprivation (Figures S59 and S60). GSEA analysis of TC@M+L showed that compared with the PBS group, mitochondrial respiration was enhanced, oxidative phosphorylation and iron‐sulfur protein assembly were also enhanced (Figures S61 and S62). Furthermore, the oxygen‐rich environment also benefits the downregulation of the HIF‐1α pathway. Glycolytic function was quantitatively assessed through Seahorse extracellular acidification rate (ECAR) assays. Compensatory glycolysis was significantly suppressed following TC@OMG+L treatment, confirming effective glycolytic inhibition (Figure 4q,r). Parallel oxygen consumption rate (OCR) measurements under sequential metabolic disturbances demonstrated elevated basal respiration, maximal respiratory capacity, ATP production, and spare respiratory capacity in the TC@OMG+L group compared to the MOF group (Figure 4s–w). These results demonstrate that TC@OMG+L treatment effectively suppressed glycolysis, prevented compensatory metabolic reprogramming, and enforced a shift toward mitochondrial oxidative phosphorylation, thereby providing a strategy targeted at increasing the sensitivity of tumor cells to cuproptosis.

### 2.9 Antitumor Effect in Subcutaneous Melanoma

The in vivo antitumor efficacy was subsequently evaluated in a subcutaneous melanoma model. The tumor mice were differentiated into several groups, including PBS, MOF, TC@OMG, and TC@OMG+L. Body weights were monitored every 3 days, with no significant weight loss observed across groups throughout the treatment period, indicating systemic tolerability of the treatments despite progressive tumor growth (Figure S63). The biodistribution profiles were intravenously injected with Cy5.5‐labeled formulations (PBS, MOF, TC@OMG). In vivo fluorescence imaging results showed transient tumor accumulation within 4 h post‐injection and rapid clearance within 12 h in the PBS group (Figure 5a,b). In contrast, both MOF and TC@OMG maintained sustained tumor fluorescence up to 24 h, with TC@OMG demonstrating significantly enhanced signal intensity as early as 12 h post‐treatment, indicating that TC@OMG can efficiently target tumor sites and achieve long‐term preservation. Meanwhile, both tumor volume and mass were reduced in the MOF and TC@OMG groups compared to the PBS group, whereas TC@OMG+L demonstrated the most potent suppression of tumor growth. This enhanced efficacy confirmed the critical role of light‐activated oxygen generation in maximizing the antitumor potential of TC@OMG (Figure 5c, Figures S64 and S65). Subsequent ICP‐MS analysis of Cu ions biodistribution revealed effective enrichment of Cu ion in tumor tissues mediated by homologous targeting (Figure 5d). Specifically, TC@OMG administration led to significantly higher intratumorally Cu accumulation compared to other groups, while concurrently reducing Cu deposition in major organs compared to MOF (Figure 5e). KEGG enrichment analysis of tumor metabolites from the MOF and TC@OMG+L treatment revealed significant enrichment of the TCA cycle and oxidative phosphorylation (Figure 5f,g). TC@OMG enhances cuproptosis via a bipartite mechanism. Firstly, the decreased local glucose concentrations trigger a compensatory elevation in GLUT1 expression. Second, the attenuated LDH‐A enzymatic activity could cause lactate reduction and Cu ions exporter MCT4 downregulation. These critical metabolic checkpoints were proved after the tumor was subjected to the treatment (Figure S66). The TC@OMG+L group exhibited the most substantial reduction in intratumorally lactate concentration, confirming effective suppression of glycolytic activity (Figure 5h).

**FIGURE 5:**
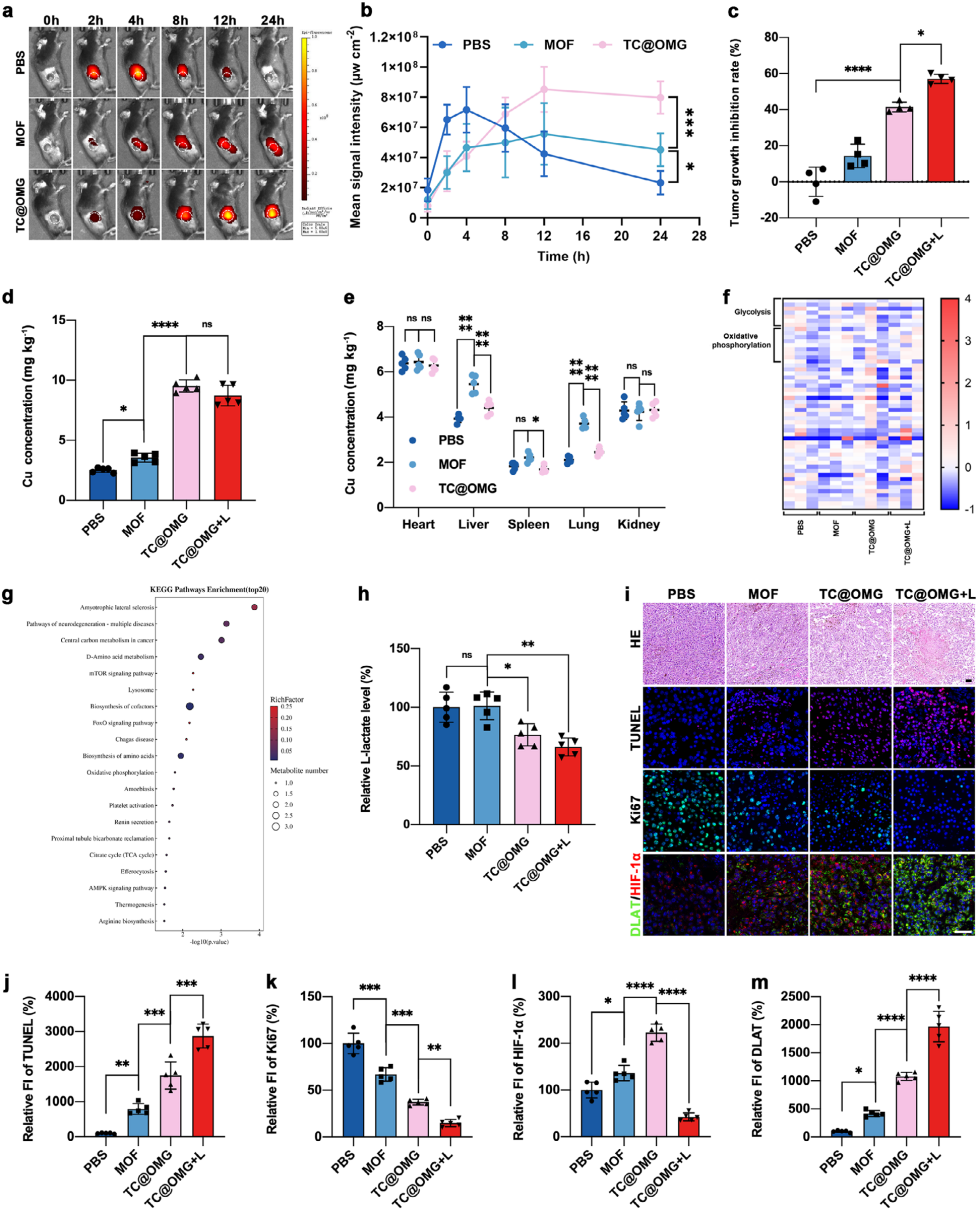
TC@OMG inhibits subcutaneous melanoma growth. (a) Drug distribution and (b) the corresponding quantification within 24 h of drug injection. Data are presented as mean ± SD. (c) Tumor growth inhibition at the treatment endpoint. (d) Total Cu levels in the tumor and (e) major organs at the treatment endpoint. (f) Metabolomics analysis of subcutaneous tumors. (g) KEGG enrichment bubble plot of metabolic features comparing TC@OMG+L‐treated and MOF‐treated B16F10 cells. (fold change ≥ 1.5, *p* < 0.05). (h) Lactate levels in primary subcutaneous melanoma after different treatment evaluated, as determined with an L‐Lactate Assay kit. (i) HE staining, TUNEL staining, Ki67 staining, and DLAT/HIF‐1α staining images of mouse tumor tissue with primary subcutaneous melanoma at the treatment endpoint. Scale bar = 50 µm. (j) Quantitative fluorescence analysis of TUNEL, (k) Ki67, (l) HIF‐1α, and (m) DLAT in tissue sections. Data are presented as mean ± SD, *n* ≥ 3. Significances are determined by one‐way and two‐way ANOVA with Tukey's correction. Significance levels are denoted as follows: *p* < 0.05(*), *p* < 0.01(**), *p* < 0.001(***), and *p* < 0.0001(****); ns, no significance.

These findings collectively demonstrated that TC membrane modification promotes tumor‐specific drug delivery while minimizing off‐target distribution. Histopathological analysis by H&E staining revealed the most significant cellular damage, consistent with the observed tumor volume inhibition in the TC@OMG+L group, surpassing all the other groups (Figure 5i). Consistent with this observation, TUNEL assays demonstrated significantly enhanced apoptotic activity, while Ki67 immunofluorescence confirmed marked suppression of cellular proliferation in the TC@OMG+L group (Figure 5j,k). These complementary histological results collectively validated the superior antitumor efficacy of TC@OMG+L treatment in subcutaneous melanoma. To explore the mechanism of tumor cell damage, the relief of hypoxic in vivo environment was assessed by HIF‐1α expression using immunofluorescence staining (Figure 5l). In the absence of irradiation, TC@OMG consumption of oxygen by GOx activity elevated HIF‐1α level. In contrast, the TC@OMG+L treatment led to a significant decrease in HIF‐1α level, indicating that light‐triggered photosynthetic oxygen generation can effectively alleviate hypoxia TME. Subsequently, the oligomerization of DLAT in tumor tissue was assessed to validate cuproptosis induction as an antitumor mechanism (Figure 5m). In summary, TC@OMG+L treatment effectively sensitized cuproptosis‐induced antitumor effect in subcutaneous melanoma through tissue Cu content increase, hypoxia relief, and glycolysis inhibition.

### 2.10 Antitumor Effect in Pulmonary Metastatic Melanoma

Therapeutic intervention for metastatic tumors presents greater challenges than for primary tumors due to their systemic dissemination and occult nature. In advanced melanoma, hematogenous spread frequently leads to pulmonary metastasis [38]. To address this clinical challenge, we further evaluated the antitumor efficacy of TC@OMG+L in an experimental pulmonary metastatic model. The treated pulmonary metastatic melanoma mice had no changed in body weight throughout the study period (Figure S67). The biodistribution characteristics of TC@OMG in the lungs were then examined. The results showed similar pharmacokinetics with primary subcutaneous tumors, with Cy5.5 fluorescence in the PBS group rapidly cleared within 12 h, while sustained pulmonary fluorescence was maintained for up to 24 h in the TC@OMG and MOF groups, with superior retention being demonstrated by TC@OMG (Figure 6a,b). At the 24‐hour time point, ex vivo lung imaging was performed, and the increased fluorescence intensity was confirmed in the TC@OMG group compared to PBS and MOF controls (Figure 6c,d). These results effectively confirmed that TC@OMG can achieve long‐term preservation and effective tumor accumulation in pulmonary metastatic melanoma. The tumors bioluminescent signal analysis revealed reduced bioluminescence intensity in both TC@OMG and TC@OMG+L groups, indicating effective suppression of lung metastasis growth (Figure 6e,f). However, no significant bioluminescence intensity difference was found between TC@OMG and TC@OMG+L treatments, which was attributed to the limited tissue penetration capability.

**FIGURE 6:**
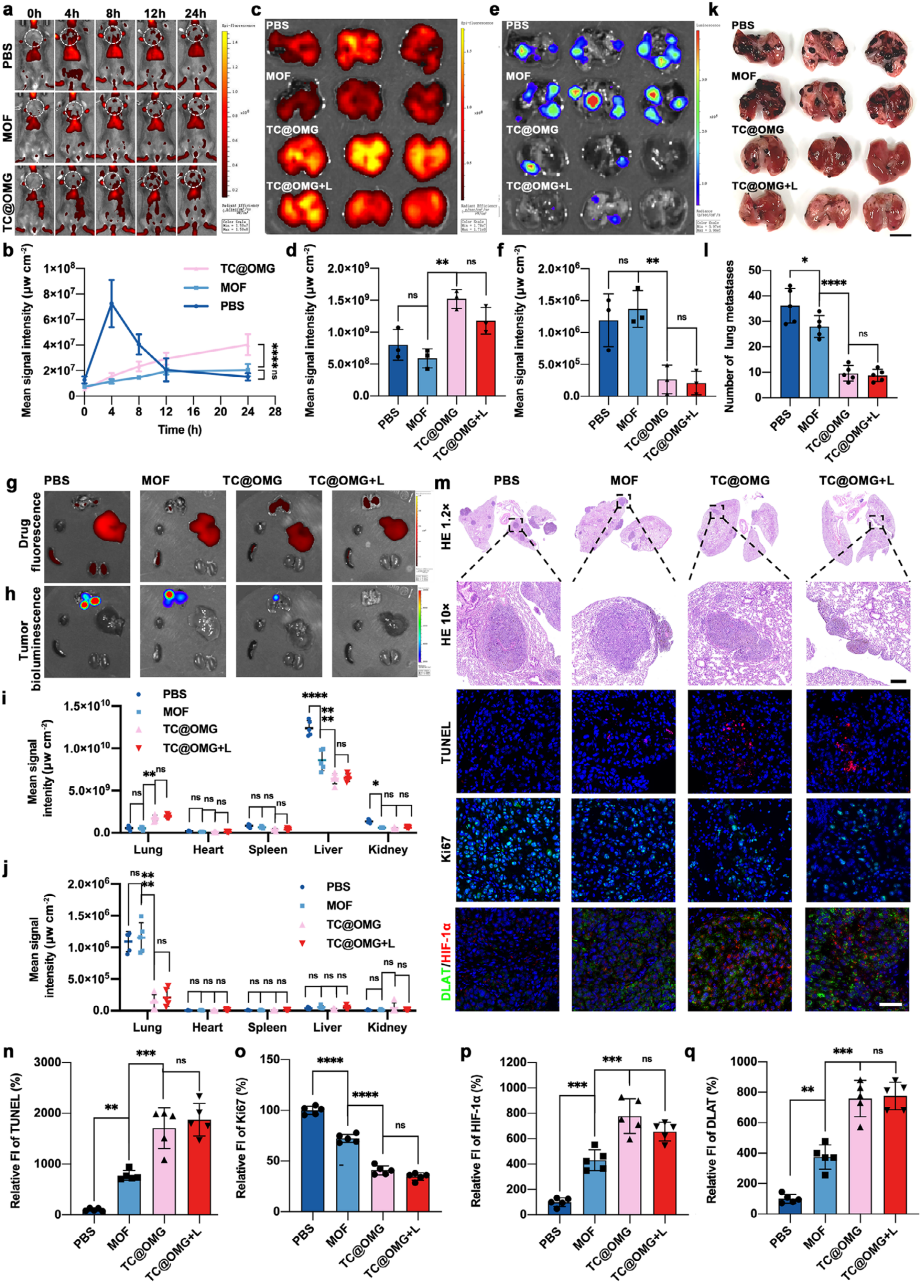
TC@OMG inhibits melanoma lung metastasis. (a) Changes in lung drug fluorescence and (b) quantification within 24 h of treatment. (c) Drug fluorescence and (d) quantification at the treatment endpoint. (e) Tumor bioluminescence and (f) quantification after different treatments. (g) Drug fluorescence and (h) tumor bioluminescence enrichment in major organs 24 h post‐treatment. (i) Drug fluorescence and (j) tumor bioluminescence enrichment quantification in major organs 24 h post‐treatment. (k) Image and (l) quantification of metastatic lung melanoma after different treatments. Scale bar = 1 cm. (m) Representative HE, TUNEL, Ki67, HIF‐1α, and DLAT staining images for tumor morphology after different treatments. Scale bar = 50 µm. (n) corresponding quantification of TUNEL, (o) Ki67, (p) HIF‐1α, and (q) DLAT staining of metastatic lung melanoma slices after different treatments. Data are presented as mean ± SD, *n* ≥ 3, significances are determined by one‐way and two‐way ANOVA with Tukey's correction. Significance levels are denoted as follows: *p* < 0.05(*), *p* < 0.01(**), *p* < 0.001(***), and *p* < 0.0001(****); ns, no significance.

The tumor‐targeting distribution capability of TC@OMG was further verified through drug fluorescent imaging of major organs. Hepatic fluorescence intensity was found to be highest in the PBS‐treated group, while significantly elevated drug fluorescence was observed in the lungs of the TC@OMG and TC@OMG+L groups (Figure 6g,i). Concurrently, pulmonary tumor bioluminescence intensity was markedly diminished in both TC@OMG and TC@OMG+L groups. Quantitative analysis of Cu content in metastatic lung melanoma confirmed significant accumulation in tumors after TC@OMG and TC@OMG+L treatments (Figure S68). Consequently, treatment with both TC@OMG and TC@OMG+L led to a significant reduction in the bioluminescence intensity of lung tumors (Figure 6h,j). The above results demonstrated that modification with TC membranes improves the nanodrug tumor targeting ability and antitumor efficacy.

Macroscopic examination of lung tissues 12 days post‐treatment revealed extensive metastatic nodule coverage in the control groups, whereas lungs treated with TC@OMG+L exhibited only 7–12 micronodules (Figure 6k,l). Histopathological analysis by H&E staining confirmed corresponding findings, with widespread metastases observed in controls compared to significantly reduced tumor burden in TC@OMG+L treated lungs (Figure 6m). These results collectively validate the significant inhibiting therapeutic efficacy of TC@OMG+L against pulmonary metastatic melanoma. TUNEL and Ki67 immunofluorescence staining results illustrated the increased tumor cell apoptosis and decreased proliferation after treatment with TC@OMG+L, confirming its enhanced antitumor efficacy (Figure 6n,o). Elevated HIF‐1α levels were also observed in both TC@OMG and TC@OMG+L groups, indicating that the limited laser's tissue penetration prevented effective photosynthetic oxygen generation in lung metastases despite GOx‐mediated glucose and oxygen consumption (Figure 6p). Furthermore, significantly enhanced DLAT fluorescence intensity was detected in the TC@OMG+L group, suggesting effective potentiation of the cuproptosis pathway (Figure 6q). These results fully demonstrated that TC@OMG+L treatment can achieve a sensitizing effect on cuproptosis‐induced antitumor effect in pulmonary metastatic melanoma through enhancing Cu enrichment, interfering glucose metabolic, and potentiating Cu^+^‐mediated cytotoxicity.

### 2.11 Antitumor Effect in Choroidal Melanoma

Subsequently, we further evaluated the therapeutic potential of TC@OMG+L for treating choroidal melanoma, which is the most common subtype of intraocular melanoma. Given the limited manipulation space and natural light‐transmitting characteristics of the ocular environment, our photoactivation platform may be well‐suited for in situ injection drug delivery. After 14 days of subretinal injection modeling and 16 days of intervention, the body weight change of the TC@OMG+L group showed no statistically significant difference from that of the control group, indicating excellent biocompatibility (Figure S69). In vivo imaging revealed that the bioluminescence intensity of tdTomato‐labeled tumors was significantly reduced in the ocular lesion area of the experimental group 16 days after administration under the retina compared to the control groups, confirming the excellent anti‐tumor performance of TC@OMG+L (Figure 7a,b). As for ocular morphology, the TC@OMG+L group exhibited a 26.77% reduction in eyeball diameter, a 50% decrease in eyeball weight, and markedly alleviated proptosis compared with the control group, demonstrating effective tumor volume control after treatment (Figure 7c,d and Figure S70). Histopathological analysis further confirmed the therapeutic effect (Figure 7e). H&E staining showed the smallest intraocular tumor volume in the TC@OMG+L group, while TUNEL staining revealed a 3.31‐fold increase in apoptotic signals (Figure 7f). Ki67 immunofluorescence indicated an 87.74% decrease in proliferation index (Figure 7g), collectively demonstrating significant suppression of tumor cell proliferation. Additionally, HIF‐1α expression was reduced by 75.89% in the TC@OMG+L group compared with the TC@OMG group (Figure 7h), with a corresponding 40.39% upregulation of its downstream target DLAT (Figure 7i). The results demonstrated that TC@OMG+L effectively suppressed intraocular tumor growth in a mouse model of choroidal melanoma, promoting tumor cell death as evidenced by upregulated TUNEL, downregulated Ki67 and HIF‐1α, and upregulated DLAT. Furthermore, this study confirmed that TC@OMG+L exerts a significant photo‐enhanced tumor‐suppressive effect within the eye. Leveraging the translucent light transmission nature of the ocular structure, TC@OMG effectively inhibited tumor proliferation and induced apoptosis.

**FIGURE 7:**
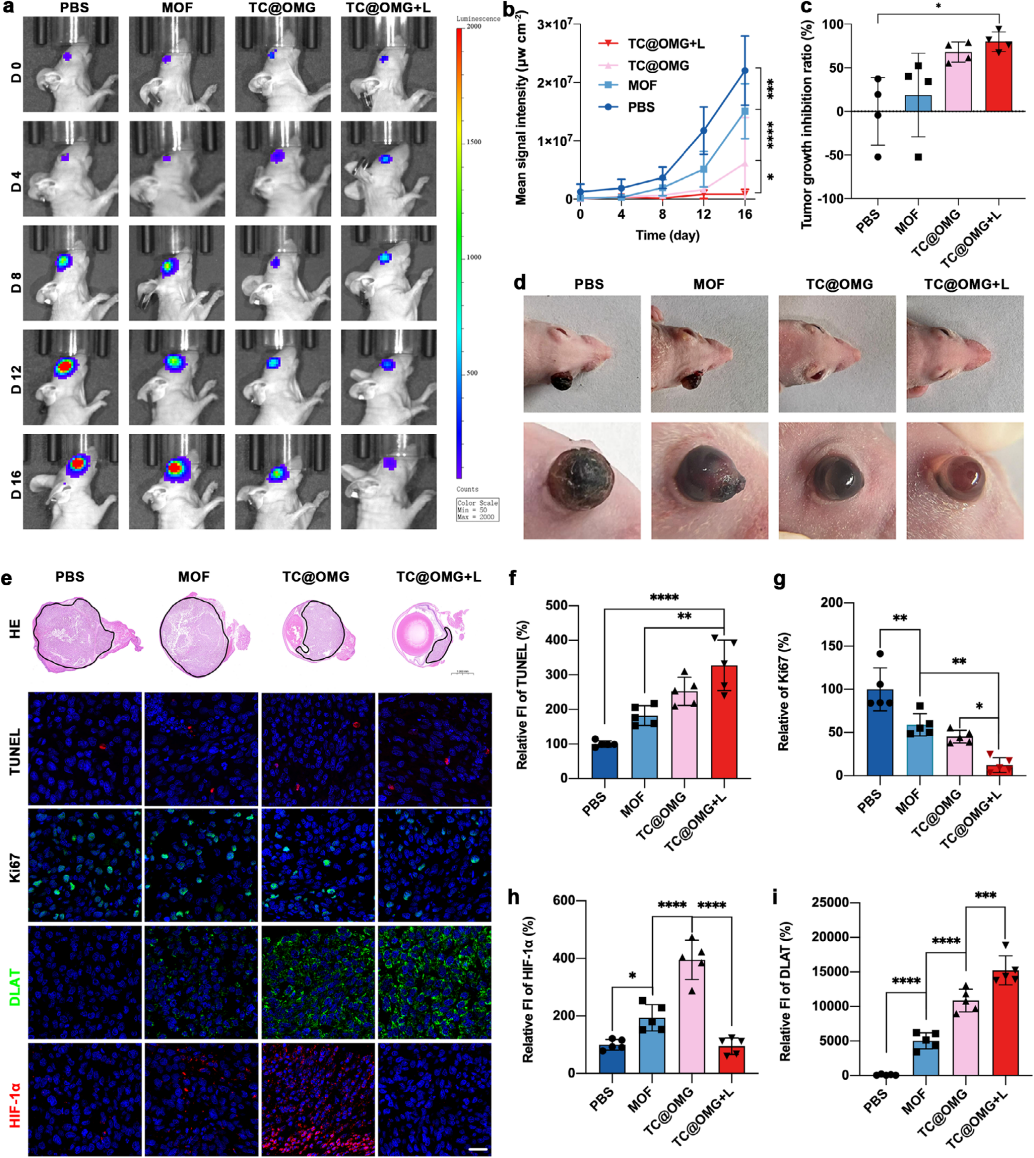
TC@OMG+L for choroidal melanoma treatment. (a) Tumor bioluminescence enrichment and the (b) quantification in eyeball after different treatments. (c)Tumor growth inhibition at the treatment endpoint. (d) Exophthalmos at the treatment endpoint. (e) HE (scale bar = 1 mm), TUNEL, Ki67, DLAT and HIF‐1α staining images of choroidal melanoma at the treatment endpoint. Scale bar = 50 µm. (f) Quantitative immunofluorescence analysis of TUNEL, (g) Ki67, (h) HIF‐1α, and (i) DLAT in choroidal melanoma after different treatments. Data are presented as mean ± SD, *n* ≥ 3. Significances are determined by one‐way and two‐way ANOVA with Tukey's correction. Significance levels are denoted as follows: *p* < 0.05(*), *p* < 0.01(**), *p* < 0.001(***), and *p* < 0.0001(****); ns, no significance.

## 3 Conclusion

A hybrid membrane‐coated Cu‐based MOF platform was reported to co‐deliver GOx and oxamic acid and enable synergistic therapy against melanoma. The Cu‐MOF degraded in response to the TME of acidic and elevated GSH levels, resulting in the rapid release of Cu^2+^ ions, which are subsequently converted to Cu^+^ to initiate cuproptosis. Mechanistic studies revealed that TK‐mediated photosynthetic oxygenation, glucose oxidase‐ and oxamic acid‐induced lactate suppression synergetic reprogrammed tumor cell respiration into mitochondrial oxidative phosphorylation, thereby increasing tumor cell susceptibility to cuproptosis. Furthermore, the nanoplatform was encapsulated by a hybrid membrane from melanoma cells and TK cells, which facilitated targeted drug delivery and photosynthetic oxygen production properties. Under laser irradiation, the system alleviated hypoxia TME by downregulating HIF‐1α, subsequently suppressed MT2A expression, and promoted mitochondrial targeting of Cu ions, further amplifying cuproptosis efficacy. The bioengineered mitochondrial respiration‐enhancing nanodrug TC@OMG demonstrated remarkable antitumor efficacy in both primary subcutaneous and choroidal melanoma models, and effectively suppresses pulmonary metastasis of melanoma. This study firstly integrated multiple hypoxia‐, metabolic reprogramming‐, and Cu homeostasis‐ targeted strategies to sensitize tumor cells in hypoxic TME to cuproptosis. This multi‐pronged nanotherapeutic lays promising potential for clinical translation.

## 4 Experimental Section

## 5 Materials

BTC, copper(II) nitrate trihydrate, cuprous chloride, triethylamine, 2,9‐Dimethyl‐1,10‐phenanthroline hemihydrate, GSH, glucose oxidase, and [(Ru(dpp)_3_]Cl_2_] were provided by Aladdin (Shanghai, China). The CCK‐8 kit was obtained from DOJINDO Laboratories (Shanghai, China). Glucose detection kit, catalase detection kit, calcein‐AM/PI kit, lactic acid test kit, ATP test kit, mitochondrial membrane potential detection kit (JC‐1), amplex red pyruvate detection kit, DCFH‐DA, DAPI, horseradish peroxidase‐labeled rabbit anti‐mouse IgG (H+L), and bicinchoninic acid (BCA) protein assay kits were purchased from Beyotime Institute of Biotechnology (Shanghai, China). RIPA lysis buffer and phosphatase inhibitor were obtained from NCM Biotechnology (Suzhou, China). Elesclomol was provided by Selleck (America). MitoTracker Green FM and cyanine 5.5 were obtained from MCE (America). Anti‐SDHB, anti‐DLST, and anti‐DLAT were provided by Proteintech (Wuhan, China).

### 5.1 Separation of Cell Membrane

The cell membrane fractions of B16F10 or 92.1 cells were isolated using a differential centrifugation method. Briefly, the cells were suspended in an ice‐cold IB‐1 buffer (225 mM mannitol, 30 mM Tris‐HCl, 75 mM sucrose, 0.5 mM EDTA, 0.5% (w/v) BSA, pH 7.4) supplemented with protease and phosphatase inhibitor cocktails (1% (v/v)). The suspension was subjected to sequential centrifugation steps: first at 800 g for 10 min at 4°C to remove intact cells and nuclei, then at 10 000 g for 20 min at 4°C. The resulting supernatant was ultracentrifuged at 100 000 g for 60 min at 4°C to pellet the cell membrane (CM). The obtained CM precipitate was lyophilized and stored at −80°C for further use.

### 5.2 Isolation of TK

Fresh spinach leaves (100 g) were washed and then homogenized in a pre‐chilled homogenizer with 300 mL of ice‐cold HEPES buffer (5 mM MgCl_2_, 50 mM NaCl, 0.3 M sucrose, pH 7.6). The homogenate was filtered through cheesecloth, and the filtrate was centrifuged at 8000 rpm for 10 min to collect the chloroplast‐enriched pellets. After washing with HEPES buffer, the chloroplast membranes were ultrasonicated on ice for 5 min. The thylakoid membranes were subsequently isolated by centrifugation at 16 000 rpm for 15 min, yielding a dark green pellet. The chlorophyll concentration was determined spectrophotometrically. The absorbances at 665 nm (A665) and 649 nm (A649) were measured, and the concentrations of chlorophyll a, b, and total chlorophyll were calculated using the following equations:

(1) Chlorophylla(μgmL−1)=13.95×A665−6.88×A649

(2) Chlorophyllb(μgmL−1)=24.96×A649−7.32×A665

(3) TotalChlorophyll(μgmL−1)=Chlorophylla+Chlorophyllb

### 5.3 Preparation and Characterization of TK‐CM Hybrid Membranes

The protein concentrations of TK and cell membrane (CM) were quantified using a BCA protein assay kit. TK and CM were blended at designated mass ratios (1:0, 3:1, 1:1, 1:3, and 0:1) and subjected to membrane fusion through sequential ultrasonication at 4°C for 10 min, followed by extrusion. The fusion process was monitored by fluorescence colocalization analysis. Specifically, TK and CM were pre‐labeled with Dio (λ_ex = 484 nm) and DiD (λ_ex = 646 nm), respectively. Fluorescence emission spectra (501‐660 nm) were acquired for each sample before and after fusion to evaluate the colocalization efficiency.

### 5.4 Synthesis of MOF

MOF was synthesized via a solvothermal method. Briefly, a solution of trimesic acid (250.0 mg) in a mixed solvent of ethanol, deionized water, and DMF (v/v/v, 1:1:1) was prepared. Separately, Cu(II) nitrate trihydrate (287.5 mg) was dissolved in an identical solvent mixture. The two solutions were then combined, followed by the slow addition of 250 mL of triethylamine. The reaction proceeded under continuous stirring at 50°C. Upon completion, the resulting blue crystals were collected by centrifugation at 13 000 rpm for 10 min. The product was thoroughly washed three times with ethanol and DMF, respectively, and subsequently dried under vacuum to afford MOF as a final product.

### 5.5 Synthesis of OMG NPs

The OMG composite was fabricated through a two‐step conjugation procedure. First, MOF (1 mL, 2 mg mL^−1^ in ethanol) was activated by mixing with a solution of EDC (1 mL, 2 mg mL^−1^ in ethanol) and NHS (0.89 µL). To this activated suspension, oxamic acid (1 mL, 1 mg mL^−1^ in DMSO) was added dropwise under stirring. The reaction mixture was stirred continuously for 6 h at room temperature. The resulting product, Oxamic‐MOF, was collected by centrifugation, washed thoroughly with ethanol, and isolated as a solid. In the subsequent step, oxamic‐MOF (1 mL, 2 mg mL^−1^) was reacted with GOx (1 mL, 2 mg mL^−1^) in ethanol under stirring for 6 h. The final OMG composite was obtained by centrifugation, followed by ethanol washes to remove unbound enzyme.

### 5.6 Synthesis of TC@OMG

The TK‐CM hybrid membrane was first prepared by blending TK (1 mL, 0.6 mg mL^−1^) and CM (1 mL, 0.6 mg mL^−1^) at a 1:1 mass ratio, followed by ultrasonication at 4°C for 10 min. Subsequently, OMG nanoparticles (1 mL, 0.2 mg mL^−1^) were introduced into the TK‐CM solution (1 mL, 0.3 mg mL^−1^) and subjected to an additional 10 min of ultrasonication to facilitate the coating process. The final TK‐CM@Oxamic‐MOF‐GOx (TC@OMG) nanoparticles were collected by centrifugation at 10 000 rpm for 5 min to remove excess membrane fragments.

### 5.7 Characterization of TC@OMG

The physicochemical properties of TC@OMG nanoparticles (1 mg mL^−1^ in aqueous solution at room temperature) were comprehensively characterized. The hydrodynamic diameter, polydispersity index (PDI), and zeta potential were measured using a Zetasizer Nano ZS90 instrument (Malvern Panalytical, UK). Morphological examination was performed with a FEI Tecnai G2 SPIRIT BioTwin transmission electron microscope (TEM). The successful coating of the cell membrane was verified by elemental mapping. The UV‐Vis absorption spectrum was recorded on a UV‐2550 spectrophotometer (Shimadzu, Japan). Fourier transform infrared (FTIR) spectroscopy was conducted on a Nicolet spectrometer (Thermo Fisher Scientific, USA). X‐ray photoelectron spectroscopy (XPS) analysis was conducted using a Shimadzu spectrometer (Japan). The crystalline phase was determined by X‐ray diffraction (XRD) on a Rigaku Ultima IV diffractometer (Japan). The chemical groups were further confirmed by ^1^H nuclear magnetic resonance (NMR) spectroscopy on a Bruker AVANCE III 600 MHz spectrometer. Elemental analysis was performed using Elementar Vario EL Cube. Thermal stability and composition were assessed by thermogravimetric analysis (TGA) on a TG209 F3 instrument (Netzsch, Germany).

### 5.8 GOx Carriers of TC@OMG

The loading efficiency of GOx onto the carriers was quantified via a colorimetric assay. Specifically, GOx was mixed with MOF or OM carriers in ethanol at varying mass ratios (GOx: Carrier = 1:10, 2:10, 3:10, 4:10, and 5:10) and stirred for 6 h. Following centrifugation, the supernatant was collected, and the concentration of unbound GOx was determined using a Bradford protein assay kit (Beyotime Biotechnology). The absorbance was measured with a UV‐Vis spectrophotometer to quantify the free GOx. The encapsulation efficiency (EE) and encapsulation capacity (EC) were calculated using the following equations:

(4) $$\begin{array}{ccc} & & \mathrm{EE}\;(\%)=\mathrm{Mass}\;\mathrm{of}\;\mathrm{drug}\;\mathrm{in}\;\mathrm{nanocarrier}/\mathrm{Mass}\;\mathrm{of}\;\mathrm{drug}\;\mathrm{fed}\;\mathrm{initially}\;\\ & & \;\times \;100\% \end{array}$$

(5) EC(%)=Massofdruginnanocarrier/Massofnanocarrier×100

### 5.9 GOx and Cu Release of TC@OMG

The cell The release profiles of Cu species and GOx from OMG and TC@OMG nanoparticles were investigated under different simulated physiological conditions. The nanoparticles were incubated for 1 h in the following four media: (i) PBS (pH 7.4), (ii) PBS (pH 5.3), (iii) PBS (pH 7.4) containing 10 mM GSH, and (iv) PBS (pH 5.3) containing 10 mM GSH. After incubation, the concentrations of the released Cu^+^, Cu^2+^, total Cu, and free GOx in the supernatant were quantitatively determined using UV‐Vis spectroscopy coupled with specific colorimetric assays. Specifically, Cu^+^ was chelated with 2,9‐dimethyl‐1,10‐phenanthroline, Cu^2+^ was chelated with sodium diethyldithiocarbamate (DDTC‐Na), total Cu was measured by inductively coupled plasma (ICP) analysis, and free GOx was quantified using the Bradford assay (Coomassie Brilliant Blue G‐250, Beyotime Biotechnology). The cumulative release curves for each component were plotted as a function of the treatment condition.

### 5.10 In Vitro GSH Detection

The GSH depletion capacity was evaluated in vitro using a DTNB assay kit (Solarbio, China). Briefly, varying concentrations of TC@OMG were incubated with 1 mM GSH solution for 30 min. The residual GSH concentration was then determined by measuring the absorbance at 412 nm according to the manufacturer's protocol. To further investigate the contribution of individual components to GSH consumption, MOF, OMG, TK‐CM, and TC@OMG (each at 100 µg mL^−1^) were separately incubated with 1 mM GSH in PBS (pH 7.4) for 30 min. After centrifugation, the remaining GSH in the supernatant was quantified using the same DTNB method.

### 5.11 Evaluation of Photosynthetic Oxygen Generation

The CAT‐like activity of the samples was assessed using a commercial Catalase Assay Kit (Beyotime Biotechnology). First, TK‐CM hybrids with different TK‐to‐CM mass ratios (1:0, 1:3, 1:1, 3:1, and 0:1) at a concentration of 2 mg mL^−1^ were incubated with 1 mM H_2_O_2_ for 30 min, after which the residual H_2_O_2_ concentration was quantified. Subsequently, the CAT‐like enzymatic activities of MOF, OMG, TK‐CM, and TC@OMG (each at 2 mg mL^−1^) were evaluated by monitoring the decomposition of H_2_O_2_ over 60 min. This catalytic performance was further corroborated by directly measuring the accompanying O_2_ generation. To investigate the operational stability of TC@OMG, the nanocatalyst was subjected to multiple reaction cycles. In each cycle, TC@OMG was incubated with 1 mM H_2_O_2_ for 1 h, followed by the replenishment of fresh H_2_O_2_ to initiate the subsequent cycle, allowing for the assessment of its sustained catalytic activity.

### 5.12 In Vitro GOx Activity Assay

The enzymatic activity of GOx was evaluated by monitoring oxygen/glucose consumption and gluconic acid/H_2_O_2_ production. MOF, OM, TC@OM, and their GOx‐loaded counterparts (GOx, OMG, TC@OMG) with equivalent GOx content were separately incubated with 10 mM glucose solution for 60 min. An additional group, TC@OMG + L, was simultaneously exposed to 660 nm laser irradiation at 400 mW cm^−2^. During the reaction, dissolved oxygen and pH values were recorded in real‐time using a portable dissolved oxygen meter and a pH meter (Lichen Technology), respectively. At designated time intervals (0, 10, 20, 30, 40, 50, and 60 min), aliquots were centrifuged, and the supernatants were collected for quantitative analysis of H_2_O_2_ and residual glucose concentrations using a commercial hydrogen peroxide assay kit and glucose assay kit (O‐toluidine method) (Beyotime Biotechnology) according to the manufacturer's protocols.

### 5.13 Cell Culture

B16F10 and 92.1 cells were maintained in RPMI‐1640 medium, while PIG1 and RPE cells were cultured in DMEM. All cell lines were incubated at 37°C in a humidified atmosphere containing 5% CO_2_, with media supplemented with 10% (v/v) fetal bovine serum and 1% (v/v) penicillin‐streptomycin.

### 5.14 Cell Uptake and Subcellular Localization

B16F10, PIG1, 92.1, and RPE cells were seeded in 24‐well plates and allowed to adhere for 12 h. The cells were then incubated with FITC‐loaded MOF and TC@M nanoparticles for 4 h. Following incubation, the cells were gently washed with PBS and fixed with 4% paraformaldehyde for 10 min at room temperature. The fixed cells were permeabilized with 0.2% Triton X‐100 for 5 min at room temperature, followed by sequential staining: actin filaments were labeled with phalloidin (Mkbio, China) for 30 min, and cell nuclei were counterstained with DAPI (Beyotime, China) for 5 min. Fluorescence images were acquired using either a ZEISS Axio Scope A1 upright microscope (Germany) or a Nikon Eclipse 80i microscope (Japan).

### 5.15 Cytotoxicity Study

B16F10 and 92.1 cells were seeded in 96‐well plates at a density of 5 × 10^3^ cells per well and allowed to adhere for 12 h at 37°C. The cells were then treated with MOF, OM, TC@M, MG, or TC@OMG at various concentrations (0, 5, 10, 20, 40, and 80 µg mL^−1^, based on MOF equivalent) for 12 h. Following treatment, the cells were either irradiated with a 660 nm laser at a power density of 400 mW cm^−2^ for 5 min or kept in the dark. After a subsequent 24‐hour incubation period, cell viability was assessed using the CCK‐8 assay according to the manufacturer's instructions.

### 5.16 Live/Dead Cell Staining Analysis

Cytotoxicity against B16F10 cells was further assessed using a Live/Dead Cell Staining Kit (Calcein‐AM/Propidium Iodide, Beyotime). Briefly, B16F10 cells were seeded in 24‐well plates and cultured for 24 h. The cells were then treated with PBS, MOF, OM, TC@M, MG, or TC@OMG at a concentration of 20 µg mL^−1^ (MOF equivalent). Following a 12‐hour incubation, the cells were either irradiated with a 660 nm laser at 400 mW cm^−2^ for 5 min or kept in the dark. After an additional 24‐hour incubation, 100 µL of Calcein‐AM/PI solution was added to each well, and the plates were incubated in the dark for 30 min. The stained cells were gently washed twice with PBS and immediately imaged using an inverted fluorescence microscope (DMi8, Leica Microsystems).

### 5.17 Colony Formation Assay

B16F10 cells were seeded in 6‐well plates at a density of 1,000 cells per well. After cell adhesion, the cultures were treated with PBS, MOF, OM, TC@M, MG, or TC@OMG (20 µg mL^−1^, MOF equivalent) for 12 h, followed by either 660 nm laser irradiation (400 mW cm^−2^, 5 min) or no light exposure. After 24 h, the medium was replaced with fresh complete medium, and the cells were cultured for an additional 1–2 weeks at 37 °C. Following the incubation period, the cells were washed with PBS, fixed with methanol, and stained with 1% crystal violet for 30 min. After removing excess dye and air‐drying, the colonies were scanned using an HP flatbed scanner and quantified with ImageJ software (Version 1.8.0, National Institutes of Health, Bethesda, MD, USA).

### 5.18 Detection of Intracellular ROS, H_2_O_2_ and GSH Levels

B16F10 cells were seeded in 24‐well plates at densities of 5 × 10^4^ cells/well (for ROS imaging) or 7.5 × 10^4^ cells/well (for H_2_O_2_ and GSH quantification) and cultured for 12 h. The cells were then treated with PBS, MOF, OM, TC@M, MG, or TC@OMG (20 µg mL^−1^, MOF equivalent) for 12 h, followed by either 660‐nm laser irradiation (400 mW cm^−2^, 5 min) or no light exposure. For ROS visualization, cells were incubated with DCFH‐DA (Beyotime, China) at 37°C for 20 min according to the manufacturer's protocol. Fluorescence images were acquired using a Nikon Eclipse 80i microscope (Japan). For H_2_O_2_ quantification, after treatments, cells were lysed by repeated freeze‐thaw cycles and centrifuged at 8000 g for 20 min at 4°C. The supernatant was collected, and the H_2_O_2_ content was measured using a Hydrogen Peroxide Assay Kit (Beyotime, China). For GSH quantification, cells were lysed by ultrasonication and centrifuged at 8000 g for 20 min at 4°C. The GSH content in the supernatant was determined using a GSH Content Assay Kit (Solarbio, China).

### 5.19 Analysis of Mitochondrial Membrane Potential

The mitochondrial membrane potential was assessed using the JC‐1 staining assay. B16F10 cells were seeded in 24‐well plates at a density of 5 × 10^4^ cells per well and cultured for 24 h. The cells were then treated with PBS, MOF, OM, TC@M, MG, or TC@OMG (20 µg mL^−1^, MOF equivalent) for 12 h, followed by either 660 nm laser irradiation (400 mW cm^−2^, 5 min) or no light exposure. Subsequently, the cells were stained with a JC‐1 probe according to the manufacturer's protocol (Mitochondrial Membrane Potential Assay Kit, Beyotime). The JC‐1 monomer was detected at excitation/emission wavelengths of 490/530 nm, while the J‐aggregates were measured at 525/590 nm. Imaging was performed using a confocal laser scanning microscope (Olympus, Japan).

### 5.20 Detection of Intracellular Oxygen Generation

Intracellular oxygen generation was monitored using an oxygen‐sensitive probe, [(Ru(dpp)_3_]Cl_2_, whose fluorescence is strongly quenched by oxygen. B16F10 cells were seeded in 24‐well plates at a density of 5×10^4^ cells per well and cultured for 24 h. The cells were then treated with PBS, MOF, OM, TC@M, MG, or TC@OMG (20 µg mL^−1^, MOF equivalent) for 1 h, followed by either 660 nm laser irradiation (400 mW cm^−2^, 5 min) or no light exposure. Subsequently, the cells were incubated with [(Ru(dpp)_3_]Cl_2_ for 4 h. Fluorescence signals (excitation: 488 nm, emission: 610 nm) were captured and recorded using a confocal laser scanning microscope (Olympus, Japan).

### 5.21 Cell Immunofluorescence Staining

Following treatment, cells were washed with PBS and fixed with 4% formaldehyde for 10 min at room temperature. The fixed cells were permeabilized with 0.2% Triton X‐100 for 5 min at room temperature and subsequently blocked with 5% BSA. Cells were then incubated with primary antibodies at 4°C overnight, followed by incubation with corresponding secondary antibodies for 60 min at room temperature. Cell nuclei were counterstained with DAPI (Beyotime) for 5 min. Digital images were acquired using a ZEISS Axio Scope A1 inverted microscope (Oberkochen, Baden‐Württemberg, Germany).

### 5.22 Transcriptome Analysis

B16F10 cells were treated with PBS, OMG, TC@M, or TC@OMG (20 µg mL^−1^, MOF equivalent) for 12 h. The TC@M and TC@OMG group were further subjected to 660 nm laser irradiation (400 mW cm^−2^, 5 min). After 24 h of incubation, total RNA was extracted using TRIzol reagent. RNA integrity was verified using the BioAnalyst 2100 system (Agilent, CA, USA). High‐throughput sequencing was performed on the Illumina Novaseq 6000 platform, followed by transcriptome analysis using StringTie software according to standard protocols. Differentially expressed genes were identified with a threshold of *p* < 0.05 and fold change ≥1.5.

### 5.23 Seahorse Assay

The oxygen consumption rate (OCR) was measured using a Seahorse XFe96 Analyzer (Agilent, USA) following the manufacturer's protocol. B16F10 cells were seeded in 24‐well plates at a density of 7.5 × 10^4^ cells per well and cultured for 12 h. The cells were then treated with MOF or TC@OMG (20 µg mL^−1^, MOF equivalent) for 12 h, followed by either 660 nm laser irradiation (400 mW cm^−2^, 5 min) or no light exposure. Immediately after treatment, the cells were trypsinized and reseeded in a Seahorse XF microplate at a density of 1 × 10^4^ cells per well. After 12 h of additional culture, OCR was measured under basal conditions and following sequential injection of oligomycin (final concentration of 1.5 µM), FCCP (final concentration of 0.5 µM), and rotenone/antimycin A (final concentration of 0.5 µM). The extracellular acidification rate (ECAR) was measured using a Seahorse XFe96 Analyzer (Agilent, USA) according to the manufacturer's instructions. B16F10 cells were seeded in 24‐well plates at a density of 7.5 × 10^4^ cells per well and cultured for 12 h. The cells were then treated with MOF or TC@OMG (20 µg mL^−1^, MOF equivalent) for 12 h, followed by either 660 nm laser irradiation (400 mW cm^−2^, 5 min) or no light exposure. Immediately after treatment, the cells were trypsinized and reseeded in a Seahorse XF microplate at a density of 1 × 10^4^ cells per well. After 12 h of additional culture, ECAR was measured under basal conditions and following sequential injection of rotenone/antimycin A (final concentration of 0.5 µM) and 2‐deoxy‐D‐glucose (final concentration of 50 mM).

### 5.24 Western Blot Analysis

Following treatment, cells were lysed using RIPA lysis buffer (NCM Biotech, China) supplemented with protease and phosphatase inhibitor cocktails. The lysates were centrifuged at 13 000 g for 30 min at 4°C to collect the protein supernatant. The proteins were denatured in SDS loading buffer by boiling, separated by 7.5% (w/v) SDS‐PAGE, and subsequently transferred to a PVDF membrane (Millipore, USA). After blocking with 5% non‐fat milk for 1 h, the membrane was incubated with primary antibodies at 4°C overnight, followed by incubation with fluorescence‐ or HRP‐conjugated secondary antibodies for 1 h at room temperature. Protein signals were visualized using Odyssey infrared imaging system (Li‐COR, USA) or chemiluminescence imaging system (Tanon, Shanghai, China).

### 5.25 In Vitro Lactic Acid, L‐DHA, Pyruvate, and ATP Detection

B16F10 cells were seeded in 24‐well plates at a density of 7.5 × 10^4^ cells per well and cultured for 12 h. The cells were then treated with PBS, MOF, OM, TC@M, MG, or TC@OMG (20 µg mL^−1^, MOF equivalent) for 12 h, followed by either 660 nm laser irradiation (400 mW cm^−2^, 5 min) or no light exposure. After 24 h, the culture supernatant was collected and centrifuged at 1000 g for 10 min at 4°C. The levels of extracellular L‐lactate and ATP in the supernatant were determined using the L‐lactate Assay Kit (WST‐8 method) and the ATP Assay Kit (Beyotime, China), respectively, according to the manufacturers’ protocols. For intracellular analysis, the cells were trypsinized, collected, and lysed by ultrasonication in an appropriate buffer. The lysates were centrifuged to obtain the supernatant, which was used to measure L‐LDH‐A (LDH) activity and pyruvate levels with the L‐Lactate Dehydrogenase Assay Kit (WST‐8 method) and the Amplex Red Pyruvate Assay Kit (Beyotime, China), respectively, following the manufacturer's instructions.

### 5.26 Biodistribution and Bioimaging

Female BALB/c nude mice were subcutaneously inoculated with B16F10 cells in the right hind flank. When the tumor volume reached 150–200 mm^3^, tumor‐bearing mice (*n* = 5 per group) were intravenously administered with free Cy5.5, or MOF and TC@OMG loaded with an equivalent dose of Cy5.5 (1 mg kg^−1^). The distribution of fluorescence was monitored at 0, 2, 4, 8, 12, and 24 h post‐injection using a near‐infrared (NIR) in vivo imaging system (Suzhou NIR Optical Technology Co., Ltd., China) with an excitation laser of 680 nm. At 24 h post‐injection, the mice were euthanized, and major organs (heart, liver, spleen, lung, kidney) and tumors were harvested for ex vivo imaging using the same NIR imaging system. The fluorescence intensity was quantified using ImageJ software.

### 5.27 In Vivo Antitumor Effect Evaluation

Female C57BL/6 mice (8 weeks old) were maintained and bred at the Experimental Animal Center of Shanghai Jiao Tong University (Shanghai, China). The study was approved by the Animal Ethics Committee of Shanghai Jiao Tong University (ethical approval number: A2025373) and was conducted in accordance with the Institutional Animal Care and Use Committee of Shanghai Jiao Tong University and the National Ministry of Health. Subcutaneous melanoma model: B16F10 cells (5 × 10^5^) suspended in 100 µL PBS were subcutaneously injected into the right hind flank of C57BL/6 mice. Five days post‐inoculation, tumor‐bearing mice (∼50 mm^3^) were randomly divided into four groups (*n* = 5 per group) and administered with PBS, MOF, or TC@OMG (0.5 mg kg^−1^, MOF equivalent) with or without laser irradiation (660 nm, 400 mW cm^−2^, 5 min). Treatments were administered intravenously on days 0, 3, and 6, with laser irradiation applied 12 h post‐injection. Tumor volume and body weight were monitored every three days, and tumor volume was calculated as (width^2^ × length)/2. On day 9, mice were euthanized, and tumors were harvested, weighed, and photographed. Lung metastasis model: B16F10 cells (2 × 10^6^) in 100 µL PBS were intravenously injected into C57BL/6 mice via the tail vein. Seven days post‐injection, mice were randomly assigned to four groups (*n* = 5 per group) and treated with PBS, MOF, or TC@OMG (0.5 mg kg^−1^, MOF equivalent) with or without laser irradiation (660 nm, 400 mW cm^−2^, 5 min). Treatments were administered on days 0, 4, and 8, with laser irradiation performed 12 h post‐injection. Body weight was monitored every four days. On day 12, mice were euthanized, and lung tissues were collected and photographed for metastasis evaluation. Choroidal melanoma model: A 1 µL suspension of 92.1 cells (1 × 10^6^ cells) previously transfected with a lentivirus expressing tdTomato was slowly intraocular injected using a microsyringe. 14 days post‐injection, the choroidal melanoma was monitored and confirmed using IVIS spectrum (PerkinElmer, USA). Subretinal therapeutic injections were administered 0, 4, 8, 12, and 16 days following successful model establishment. The injected drugs were 1 µL of PBS, MOF, or TC@OMG (all at a concentration of 0.5 mg mL^−1^, calculated based on MOF equivalence), followed immediately by laser irradiation. The body weight was monitored every 4 days. The mice were euthanized on day 16, and then the ocular tissues were collected.

### 5.28 H&E, TUNEL, and Immunofluorescence Staining of Tissues

Tumor tissues were fixed in 4% paraformaldehyde, embedded in paraffin, and sectioned. For H&E and TUNEL staining, sections were deparaffinized, rehydrated, and stained following standard procedures or the TUNEL kit protocol. For immunofluorescence, after deparaffinization and rehydration, antigen retrieval was performed using antigen retrieval buffer. Sections were blocked with 3% bovine serum albumin for 30 min, incubated with primary antibodies at 4°C overnight, incubated with Cy3‐ or FITC‐conjugated secondary antibodies in the dark for 1 h, and counterstained with DAPI for 10 min. Slides were scanned using an Aperio ScanScope CS scanner (Leica) and analyzed with CaseViewer v2.4 software (3DHISTECH).

### 5.29 Metabolomics Analysis

Metabolites were extracted from animal tissues using 80% (v/v) aqueous methanol. The extracts were subjected to ultrasonication, followed by centrifugation and evaporation under a gentle nitrogen stream for derivatization. Standard solutions were prepared by sequential dilution, mixed with quality control samples and sucrose alcohol, and then dried under nitrogen. Analysis was performed using an Agilent 7890A gas chromatography system coupled with an Agilent 5975C inert MSD system, employing a specific chromatographic column, helium carrier gas, and controlled temperature program for separation. Data were acquired in selected ion monitoring mode and quantified using MSD ChemStation software.

### 5.30 In Vivo Biosafety Assessment

Healthy C57BL/6 mice were intravenously administered with saline, MOF, or TC@OMG (0.5 mg kg^−1^, MOF equivalent). Body weight was recorded every three days. After 12 days, the mice were euthanized, and blood samples were collected for complete blood panel and biochemical analysis. Major organs (heart, liver, spleen, lungs, and kidneys) were harvested for histological examination.

### 5.31 Statistical Analysis

Statistical analyses were performed using GraphPad Prism 8.0.2 (GraphPad Software, San Diego, CA, USA). Data are presented as the mean ± standard deviation (SD) unless otherwise specified. For comparisons between two groups, a two‐tailed Student's t‐test was applied. For multiple group comparisons, one‐way analysis of variance (ANOVA) with Tukey's correction was used as appropriate. Significance levels are denoted as follows: *p* < 0.05(*), *p* < 0.01(**), *p* < 0.001(***), and *p* < 0.0001(****).

## Author Contributions

**Liyang Zhou**: funding acquisition, data curation, supervision, resources, project administration, Writing – review and editing. **Hongpei Deng**: conceptualization, investigation, writing – original draft, methodology, validation, visualization, data curation. **Tingli Zheng**: validation, methodology, visualization, software, formal analysis. **Huan Min**: methodology, validation, visualization, software, formal analysis. **Qiwei Tian**: methodology, validation, visualization, software, formal analysis. **Huimin Lin**: methodology, validation, visualization, software, formal analysis. **Jing Ruan**: funding acquisition, data curation, supervision, resources, project administration, writing – review and editing. **Xianqun Fan**: funding acquisition, writing – review and editing, project administration, data curation, supervision, resources.

## Funding

This work was supported by the National Natural Science Foundation of China (82388101, 82371103), the Fundamental and Interdisciplinary Disciplines Breakthrough Plan of the Ministry of Education of China (JYB2025XDXM611), the Open Project of Translational Medicine of SJTU (No. TMSK‐2024‐302), and the Doctoral Innovation and Training Fund of Shanghai Jiao Tong University School of Medicine (24KCPYYB013).

## Conflicts of Interest

The authors declare no conflicts of interest.

## Supporting information




**Supporting File**: advs78669‐sup‐0001‐SuppMat.docx.

## Data Availability

All data are available in the main text or the supplementary materials.
