# Supplementary material for: A Bioengineered Mitochondrial Respiration‐Mimetic Copper‐Based Metal‐Organic Framework Reprogramming Oxidative Phosphorylation to Enhance Tumor Cuproptosis
Source: Adv Sci (Weinh). 2026 Nov 9:e78669. Online ahead of print. doi: 10.1002/advs.78669 (PMC13748765; doi:10.1002/advs.78669)
Supplement: Supplementary file 1 [file ADVS-9999-e78669-s001.docx]

This Supporting Information file includes: TOC Figure and Figsure S1 to S70

A Bioengineered Mitochondrial Respiration-mimetic Copper-based Metal Organic Framework Reprogramming Oxidative Phosphorylation to Enhance Tumor Cuproptosis

TOC Figure：

Supporting Information

A Bioengineered Mitochondrial Respiration-mimetic Copper-based Metal Organic Framework Reprogramming Oxidative Phosphorylation to Enhance Tumor Cuproptosis

Liyang Zhou^a#^, Hongpei Deng^a#^, Tingli Zheng^a#^, Huan Min^b#^, Qiwei Tian^c^, Huimin Lin^a^, Jing Ruan^a^*, Xianqun Fan^a^*

a. Liyang Zhou, Hongpei Deng, Tingli Zheng, Huimin Lin, Jing Ruan, Xianqun Fan

State Key Laboratory of Eye Health, Shanghai Key Laboratory of Orbital Diseases and Ocular Oncology, Department of Ophthalmology, Ninth People’s Hospital, Shanghai Jiao Tong University School of Medicine, Shanghai, 200011, P. R. China.
E-mail: drjruan@shsmu.edu.cn; fanxq@sjtu.edu.cn

b. Huan Min
Henan Institute of Advanced Technology, Zhengzhou University, Zhengzhou, China.

c. Qiwei Tian

Shanghai Key Laboratory of Molecular Imaging, Shanghai University of Medicine and Health Sciences, Shanghai, China.

Liyang Zhou, Hongpei Deng, Tingli Zheng, and Huan Min contributed equally to this work.

**Figure S1.** Lactate and pyruvate concentrations in B16F10 cells after different treatments. Data are expressed as mean ± SD, *n* = 3.

**Figure S2.** Lactate and pyruvate concentrations in 92.1 cells after different treatments. Data are expressed as mean ± SD, *n* = 3.

**Figure S3.** Synthesis route of TC@OMG.

**Figure S4.** The FT-IR spectrum of MOF.

**Figure S5.** XRD pattern of MOF.

**Figure S6.** TEM image of MOF.

**Figure S7.** TEM image of TC@OMG.

**Figure S8.** XPS spectra of N1s of OMG.

**Figure S9.** a) ^1^H MR spectrum of MOF and b) oxamic acid.

**Figure S10.** ^1^H MR spectrum of OM at 25 and 80℃.

**Figure S11.** Elemental analysis (EA) of the MOF and OM. Data are expressed as mean ± SD.

**Figure S12.** XPS spectra analysis of Cu 2p orbit.

**Figure S13.** N_2_ adsorption-desorption isotherms of MOF, OM, and OMG.

**Figure S14.** UV-vis absorbance of G250, GOx, and GOx + G250.

**Figure S15.** Encapsulation capacity (blue) and encapsulation efficiency (orange) of GOx in MG at varying GOx/MOF feed ratio. Data are expressed as mean ± SD, *n* = 3.

**Figure S16.** Encapsulation capacity (blue) and encapsulation efficiency (orange) of GOx in OMG at varying GOx/OM feed ratio. Data are expressed as mean ± SD, *n* = 3.

**Figure S17.** Confocal fluorescence microscopy analysis to visualize membrane fusion. Scale bar = 5 μm.

**Figure S18.** Thermogravimetric analysis of TC, OMG, and TC@OMG.

**Figure S19.** UV-vis absorbance of CuCl_2_, DDTC-Na, and DDTC-Na + CuCl_2_.

**Figure S20.** Acid- and GSH-responsive Cu^2+^ release ability of MOF. Data are expressed as mean ± SD, *n* = 3.

**Figure S21.** Acid- and GSH-responsive Cu^+^ release ability of MOF. Data are expressed as mean ± SD, *n* = 3.

**Figure S22.** UV-vis absorbance spectra of DDTC-Na by PBS (pH = 5.3) and different concentrations of TC@OMG (0 ~ 200 μg mL^-1^).

**Figure S23.** UV-vis absorbance spectra of neocuproine by GSH (10 mM) and different concentrations of TC@OMG (0 ~ 200 μg mL^-1^).

**Figure S24.** UV-vis absorbance spectra of DTNB by GSH (1 mM) and different concentrations of TC@OMG (0 ~ 200 μg mL^-1^).

**Figure S25.** Acid- and GSH-responsive GOx release ability of MOF. Data are expressed as mean ± SD, *n* = 3.

**Figure S26.** CAT ability of different TK: CM ratios to catalyze 1 mM H_2_O_2_, *n* = 3.

**Figure S27.** CAT ability of MOF, TC, OMG, and TC@OMG to catalyze 1 mM H_2_O_2_ in 60 min, *n* = 3.

**Figure S28.** Oxygen production after MOF, OMG, TK, TC, and TC@OMG catalysis H_2_O_2_ in 180 s, *n* = 3.

**Figure S29.** Oxygen production of different ratios of TK-CM irradiated with 660 nm laser for 10 min, *n* = 3.

**Figure S30.** Oxygen production of TC@OMG, T@OMG, C@OMG, and OMG after 660 nm laser irradiation for 10 min. Data are expressed as mean ± SD, *n* = 3.

**Figure S31.** Oxygen production of different TC@OMG concentrations, *n* = 3.

**Figure S32.** Oxygen production of different irradiation fluxes, *n* = 3.

**Figure S33.** Glucose-responsive GOx release ability of TC@OMG. Data are expressed as mean ± SD, *n* = 3.

**Figure S34.** Glucose-responsive Cu^2+^ release ability of TC@OMG. Data are expressed as mean ± SD, *n* = 3.

**Figure S35.** Glucose-responsive Cu^+^ release ability of TC@OMG. Data are expressed as mean ± SD, *n* = 3.

**Figure S36.** Relative viability of cells after incubation with GOx at various concentrations for 24 h. Data are expressed as mean ± SD, *n* = 3. Bule: B16F10 cells, red: 92.1 cells.

**Figure S37.** Relative viability of cells after incubation with oxamic acid at various concentrations for 24 h. Data are expressed as mean ± SD, *n* = 3. Bule: B16F10 cells, red: 92.1 cells.

**Figure S38.** Relative viability of cells after incubation with TC at various concentrations for 24 h. Data are expressed as mean ± SD, *n* = 3. Bule: B16F10 cells, red: 92.1 cells.

**Figure S39.** a) B16F10 cells stained with Calcein-AM/PI after TC@OMG treatments and b) the corresponding quantitative analysis. Calcein-AM (green) represents live cells, and PI (red) represents dead cells. Scale bar = 100 μm, *n* = 3.

**Figure S40.** a) Representative images of the colony formation assay to assess B16F10 cell growth after TC@OMG treatments and b) the corresponding quantitative analysis, *n* = 3.

**Figure S41.** Viability of PIG1 cells after incubation with MOF and TC@M at various concentrations for 24 h. Data are expressed as mean ± SD, *n* = 3.

**Figure S42.** Viability of RPE cells after incubation with MOF and TC@M at various concentrations for 24 h. Data are expressed as mean ± SD, *n* = 3.

**Figure S43.** Relative viability of PIG1 and RPE cells after incubation with TC@OMG at various concentrations for 24 h. Data are presented as mean ± SD, *n* = 3.

**Figure S44**. a) B16F10 cells stained with Calcein-AM/PI after TK, CM, and TC treatments and b) the corresponding quantitative analysis. Calcein-AM (green) represents live cells, and PI (red) represents dead cells. Scale bar = 100 μm. Data are expressed as mean ± SD, *n* = 3.

**Figure S45.** The hemolysis assays of different treatments and the corresponding photographs. Data are expressed as mean ± SD, *n* = 3.

**Figure S46.** The hemolysis assays of 660 nm laser with TC@OMG at different concentrations and the corresponding photographs. Data are expressed as mean ± SD, *n* = 3.

**Figure S47.** Biocompatibility evaluation of TC@OMG after tail vein injection. a) Blood routine, b) biochemistry results, and c) major organ HE observation after PBS, MOF, and TC@OMG treatments. Scale bar = 100 μm.

**Figure S48.** Body weight changes in non-tumor-bearing normal mice following different treatments. Data are expressed as mean ± SD, *n* = 5.

**Figure S49.** a) Western blot images of NDUFB8 and FDX1 relative to ACTIN in B16F10 cells after different treatments. b) Relative intensities of NDUFB8 and FDX1 normalized to ACTIN. Data are presented as mean ± SD, n = 3.

**Figure S50.** a) Intracellular hypoxia levels and b) quantification using RDPP probe after different treatments. Scale bar = 100 μm. Data are expressed as mean ± SD, *n* = 5.

**Figure S51.** H_2_O_2_ concentration in the supernatant of B16F10 cells after different treatments, as determined with a hydrogen peroxide assay kit. Data are expressed as mean ± SD, *n* = 3.

**Figure S52.** Glucose concentration in the supernatant of B16F10 cells after different treatments, as determined with a glucose kit. Data are expressed as mean ± SD, *n* = 3.

**Figure S53.** L-lactate concentration in the supernatant of B16F10 cells after different treatments, as determined with an L-lactate Assay kit. Data are expressed as mean ± SD, *n* = 3.

**Figure S54.** The L-lactate dehydrogenase levels in B16F10 cells under different treatments were evaluated using an L-lactate dehydrogenase assay kit. Data are expressed as mean ± SD, *n* = 3.

**Figure S55.** Variation among samples described using principal component analysis (PCA) of gene expression.

**Figure S56.** The correlation between samples was shown by inter-sample Pearson correlation analysis.

**Figure S57.** Volcano plots using RNA-seq analysis depict different expressed genes between the TC@OMG+L and PBS groups.

**Figure S58.** GSEA showing that the mountain plot for P53 pathway activation marker genes was enriched in B16F10 cells treated with TC@OMG+L.

**Figure S59.** a) GO enrichment analysis and b) KEGG pathway enrichment analysis of different expressed genes between the OMG and PBS groups.

**Figure S60.** GSEA showing that the mountain plot for a) cell cycle, b) apoptosis activation, c) P53 pathway, and d) HIF-1α pathway marker genes were enriched in B16F10 cells treated with OMG.

**Figure S61.** a) GO enrichment analysis of different expressed genes between the TC@M+L and PBS groups and b) KEGG pathway enrichment analysis.

**Figure S62.** GSEA showing that the mountain plot for a) oxidative phosphorylation, b) mitochondrial ATP synthesis, c) aerobic respiration, d) iron-sulfur protein assembly, e) pyruvate metabolism, f) mitochondrial electron transport processes, g) respiratory electron transport chain, and h) HIF-1α pathway marker genes were enriched in B16F10 cells treated with TC@M+L.

**Figure S63**. Body weight of B16F10 tumor-bearing mice after different treatments for primary subcutaneous melanoma. Data are expressed as mean ± SD, *n* = 5.

**Figure S64.** a) Tumor morphology changes and b) tumor weight at the treatment endpoint. Scale bar = 1 cm, *n* = 4.

**Figure S65.** Tumor volume change during the treatment, *n* = 4.

**Figure S66.** a) Representative fluorescence images and corresponding quantitative b) GLUT1 staining, c) LDH-A staining, and d) MCT4 staining fluorescence analysis of mouse tumor tissue with primary subcutaneous melanoma at the treatment endpoint. Scale bar = 50 μm. Data are presented as mean ± SD, *n* = 5.

**Figure S67.** Body weight of B16F10 tumor-bearing mice after different treatments for metastatic lung melanoma. Data are expressed as mean ± SD, *n* = 5.

**Figure S68.** The Cu concentration in metastatic lung melanoma after different treatments detected by ICP-MS. Data are expressed as mean ± SD, *n* = 5.

**Figure S69.** Body weight in mice with choroidal melanoma after different treatments. Data are expressed as mean ± SD, *n* = 5.

**Figure S70. a**) Morphological changes, b) eyeball diameter, and c) weight at the treatment endpoint. Scale bar = 1 cm, *n* = 4.
